# Developmental changes in myocardial B cells mirror changes in B cells associated with different organs

**DOI:** 10.1172/jci.insight.139377

**Published:** 2020-08-20

**Authors:** Cibele Rocha-Resende, Wei Yang, Wenjun Li, Daniel Kreisel, Luigi Adamo, Douglas L. Mann

**Affiliations:** 1 Center for Cardiovascular Research, Cardiovascular Division, Department of Medicine,; 2 Genome Technology Access Center, Department of Genetics,; 3 Department of Surgery, and; 4 Department of Pathology and Immunology, Washington University School of Medicine, St. Louis, Missouri, USA.

**Keywords:** Cardiology, Immunology, B cells

## Abstract

The naive heart harbors a population of intravascular B cells that make close contact with the cardiac microvasculature. However, the timing of their appearance and their organ specificity remain unknown. To address this knowledge gap, we performed a systematic analysis of B cells isolated from the myocardium and other organs, from embryonic life to adulthood. We found that the phenotype of myocardial B cells changed dynamically during development. While neonatal heart B cells were mostly CD11b^+^ and CD11b^–^ CD21^–^CD23^–^, adult B cells were predominantly CD11b^–^CD21^+^CD23^+^. Histological analysis and intravital microscopy of lung and liver showed that organ-associated B cells in contact with the microvascular endothelium were not specific to the heart. Flow cytometric analysis of perfused hearts, livers, lungs, and spleen showed that the dynamic changes in B cell subpopulations observed in the heart during development mirrored changes observed in the other organs. Single cell RNA sequencing (scRNAseq) analysis of B cells showed that myocardial B cells were part of a larger population of organ-associated B cells that had a distinct transcriptional profile. These findings broaden our understanding of the biology of myocardial-associated B cells and suggest that current models of the dynamics of naive B cells during development are incomplete.

## Abstract 2

Myocardial B cells are part of a larger population of intravascular organ-associated B cells.

## Introduction

The immune system plays a critical role in the context of myocardial homeostasis and adaptation to injury (1), and it is therefore critical to understand the biology of the myocardial leukocyte pool. Surprisingly, while B cells are the second most prevalent leukocyte population in the naive murine heart (2, 3) and play important roles in the context of cardiac adaptation to injury (4–9), little is known with respect to the basic aspects B cell biology in the heart. Current models of B cell biology posit that, in the absence of tissue inflammation, B cells continuously circulate through blood and lymphatic vessels, moving between primary and secondary lymphoid tissue without accumulating in peripheral tissue (10–12).

However, this prevailing view has been challenged by the notion that myocardial-associated B cells are a subset of circulating B cells. We have recently identified a population of intravascular myocardial-associated B cells that remain in close approximation with the microvascular endothelial cells in the heart, in the absence of discernible tissue injury, and express a transcriptional profile that is distinct from that of circulating peripheral B cells (13). Our initial observations were limited to characterizing B cell biology in the naive adult mouse heart and did not address more fundamental questions regarding the characteristics of B cells in the embryonic, neonatal, and adult heart — nor did they address whether myocardial B cells shared common biological profiles with B cells in other organs. In the present study, we characterize myocardial B cells from embryonic to early adult life and compare these changes with those occurring in B cells in the blood, spleen, liver, and lung using a combination of flow cytometry, immunofluorescence, intravital microscopy, and single cell RNA sequencing (scRNAseq). Here, we show for the first time to our knowledge that myocardial-associated B cells are in equilibrium with splenic B cells throughout life and are representative of a much larger population of B cells that are associated with the lung and liver.

## Results

### Characterization of myocardial-associated B cells from embryonic to adult stage.

We have previously described (5, 13) 3 subsets of CD19^+^ B cells in the murine heart: (a) CD11b^+^CD5^+^IgM^hi^, consistent with B1a cells; (b) CD11b^+^CD5^–^IgM^hi^, consistent with B1b cells; and (c) CD11b^–^ B2 cells. By further refining our tissue digestion protocol, we were able to reliably identify 2 additional CD markers frequently used to classify murine B cells, namely CD21 and CD23 (14) (Supplemental Figure 1; for details, see Methods; supplemental material available online with this article; https://doi.org/10.1172/jci.insight.139377DS1). Using this optimized protocol, we identified 2 additional CD19^+^CD11b^–^ myocardial B cell subsets: (a) CD21^+^CD23^+^ and (b) CD21^–^CD23^–^, which are characteristic of multiple subtypes of B2 cells (full gating strategy and myocardial subsets are shown in Supplemental Figure 2).

**Figure 1:**
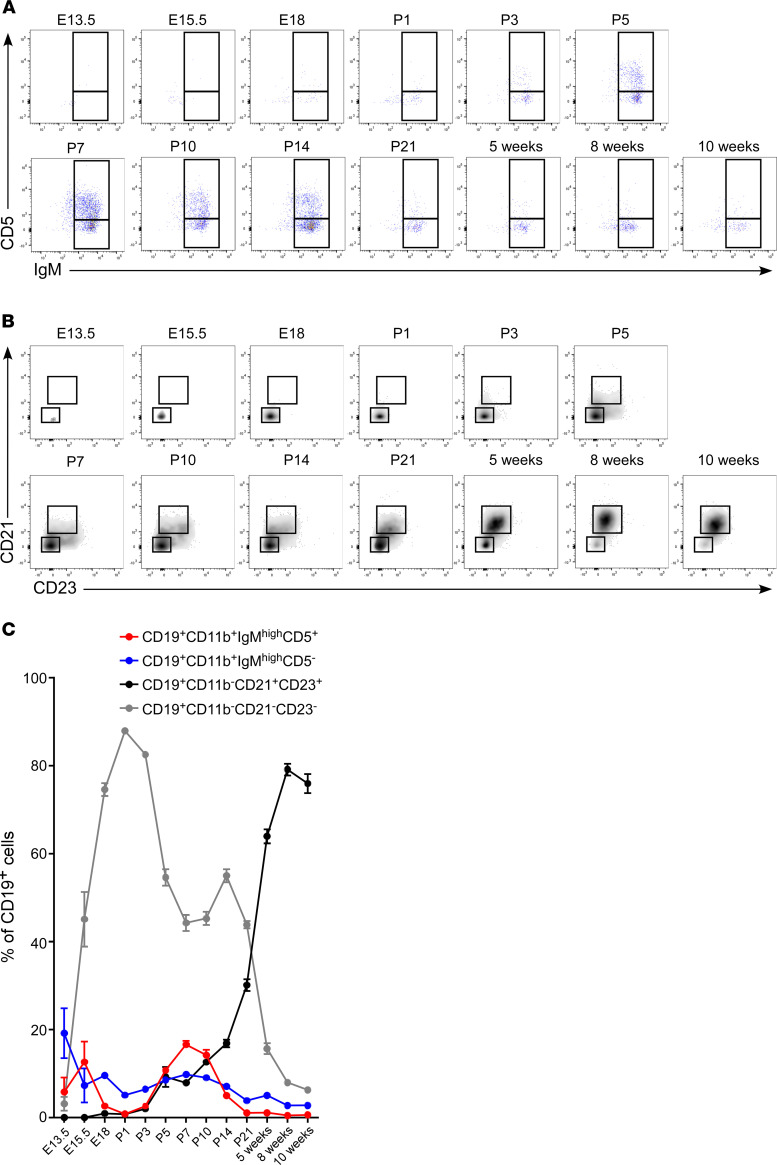
Myocardial B cell subsets change from late embryonic life to adulthood. (**A**) Representative flow charts showing the expression of CD5 and IgM in CD19^+^CD11b^+^ B cells in the heart from E13.5 to 10 weeks of age. The box inserts within each graph delineate the positions of the IgM^hi^CD5^+^ subset and of the IgM^hi^CD5^–^ subset. The prevalence of IgM^hi^CD5^+^ B cells increases during neonatal life (peak at P7), but this is not sustained during adult life. (**B**) Representative flow charts of CD21 and CD23 expression in myocardial CD19^+^CD11b^–^ B cells. The box inserts within each graph delineate CD21^–^CD23^–^ and CD21^+^CD23^+^ subsets. CD21^–^CD23^–^ cells are the majority of B cells at birth, and their prevalence gradually decreases as the prevalence of CD21^+^CD23^+^ increases. CD21^+^CD23^+^ constitute the major B cell subgroup in the adult naive heart. (**C**) Summary graph showing the dynamic changes in B cell composition from embryonic to adult life in the heart. *n* = 4–7 samples. Supplemental Table 1 shows the statistical analysis of each subset from embryonic through adult life. From E13.5 to P7, 3–6 embryonic and neonatal hearts were pooled together to constitute *n* = 1.

In order to study the characteristics of B cells in the heart, we analyzed freshly isolated hearts of naive WT mice from E13.5 to 10 weeks of age. We found that B cells were present in the heart at E13.5 and that the relative prevalence of myocardial B cell subsets changed dynamically during development and early postnatal life (Figure 1, A–C). CD19^+^CD11b^+^IgM^hi^CD5^+^ cells were more abundant in the embryonic hearts (E13.5–E15.5). Their prevalence decreased rapidly by the time of birth (P1) but increased again in early neonatal life (peak at P7) (Figure 1A and Supplemental Table 1). This increase was not sustained, however, and this subgroup of B cells constituted only a small fraction of B cells in the adult heart (Figure 1 and Supplemental Table 1). At birth, most myocardial B cells were CD19^+^CD11b^–^CD21^–^CD23^–^. The prevalence of this subpopulation gradually decreased as the mice aged, whereas the prevalence of the CD19^+^CD11b^–^CD21^+^CD23^+^ subset increased (Figure 1 and Supplemental Table 1). The expression of IgM and IgD in CD11b^–^ B cells also changed dynamically during development (Supplemental Figure 3), with IgM^hi^IgD^lo^ subsets of B cells prominent in embryonic and P1 through P14 hearts, and IgM^hi^IgD^hi^ and IgM^lo^IgD^hi^ B cells prominent during late postnatal (P21) and adult hearts.

**Figure 2:**
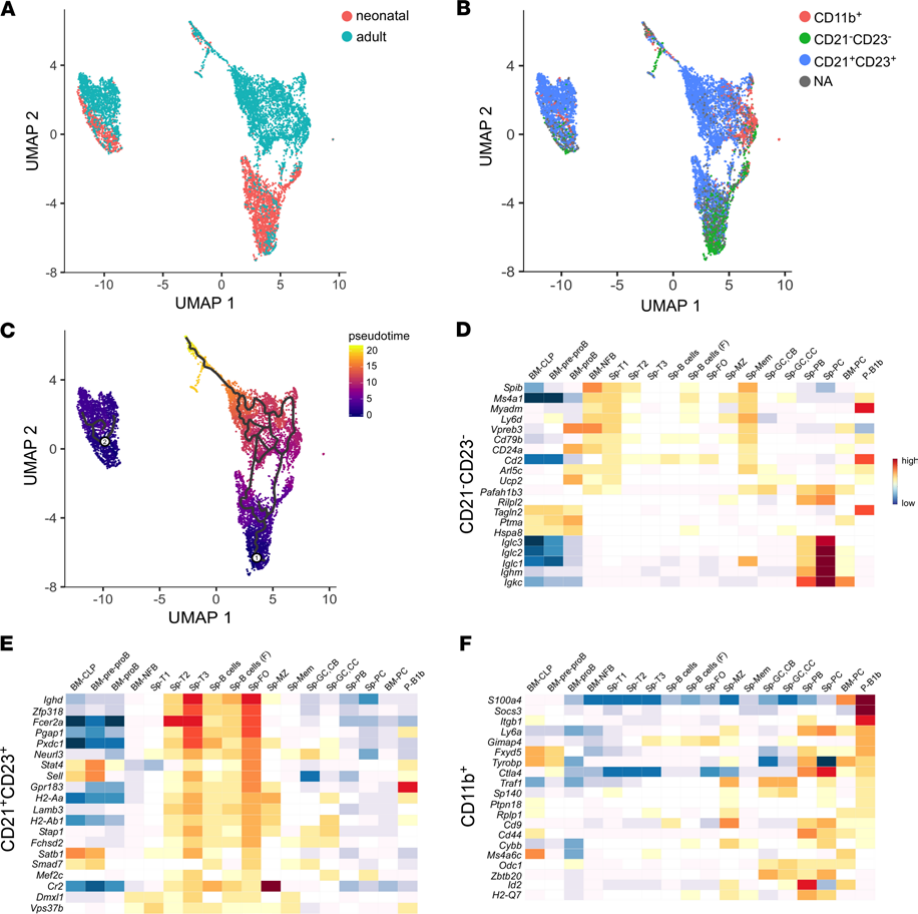
Transcriptional profiling identifies myocardial B cells as a heterogeneous, dynamic population of transitional, follicular, and B1 cells. (**A**) A 10× sequencing analysis of CD45^+^Aqua^–^CD19^+^ cells sorted from the heart of neonatal (2 week old) and adult (8 week old) mice. Neonatal and adult cardiac B cells show a distinct transcriptional profile. (**B**) Subsets of B cells from neonatal and adult myocardium. Cardiac B cells were stained with TotalSeq antibodies for CD11b, CD23, and CD21 before sequencing. Comparison of this UMAP plot with the UMAP plot reported in **A** shows that CD21^+^CD23^+^ cells are mostly found in the adult heart, while CD21^–^CD23^–^ are mostly neonatal. (**C**) Differentially expressed genes between B cell subsets were used to generate hypothetical developmental relationships using Monocle algorithms. Pseudotime analysis indicates that CD21^–^CD23^–^ cells move toward CD21^+^CD23^+^ cells. (**D–F**) Heatmaps reporting the relative expression of the top 20 unique upregulated genes in the CD21^–^CD23^–^ (**D**), CD21^+^CD23^+^ (**E**), and CD11b^+^ (**F**) myocardial cells within various B cell subtypes catalogued in the Immgen RNAseq database (for details, see Supplemental Table 2). The transcriptional profile of CD21^+^CD23^+^ myocardial B cells resembles the transcriptional profile of splenic Transitional 3 (T3) and follicular cells (**D**). Cardiac CD21^–^CD23^–^ cluster are similar to T1 and newly formed B cells (BM-NFB) (**E**). CD11b^+^ myocardial B cells are transcriptionally similar to B1 cells in the peritoneal cavity (**F**). Sp, spleen; P, peritoneal; CLP, common lymphoid progenitor; NFB, newly formed B cell; T, transitional; (F), female; FO, follicular; MZ, marginal zone; Mem, memory; GC, germinal center; CB, centroblasts; CC, centrocytes; PB, plasmasblasts; PC, plasma cells.

In order to gain further insight into the identity of the various myocardial B cells subsets, we performed scRNAseq of neonatal (2 weeks) and adult (8 weeks) myocardial B cells (Figure 2). We combined 10× single cell gene expression analysis with immunostaining using TotalSeq antibodies against CD11b, CD23, and CD21 (Supplemental Table 10). Heart B cells sorted from neonatal mice showed a distinct gene expression profile in comparison with B cells sorted from the adult heart (Figure 2A) and were mostly CD21^–^CD23^–^ (Figure 2B), whereas in the adult heart, B cells were mostly CD21^+^CD23^+^ (Figure 2B). To assess the relationship between CD21^+^CD23^+^ and CD21^–^CD23^–^ cells, we performed a pseudotime analysis using the density of CD21^–^CD23^–^ cells to guide the pseudotime estimation. This analysis showed a trajectory from CD21^–^CD23^–^ cells in neonatal hearts to CD21^+^CD23^+^ cells in adult hearts (Figure 2C), suggesting that CD21^–^CD23^–^ B cells mature into CD21^+^CD23^+^ B cells. Comparison of the genes uniquely upregulated in CD21^–^CD23^–^ cells, CD21^+^CD23^+^ cells, and CD11b^+^ cells using the Immgen RNAseq signature database identified myocardial CD21^–^CD23^–^ cells as newly formed B cells (NFB)/transitional 1 (T1) cells (Figure 2D), myocardial CD21^+^CD23^+^ B cells as T3/follicular (FO) cells (Figure 2E), and CD11b^+^ cells as B1 cells (Figure 2F) (Supplemental Table 2). Viewed together, these analyses suggest that myocardial B cells are composed of subsets of follicular, transitional, and B1 cells, and that the ratio between these different subtypes of B cells changes dynamically from embryonic life to adulthood.

**Figure 3:**
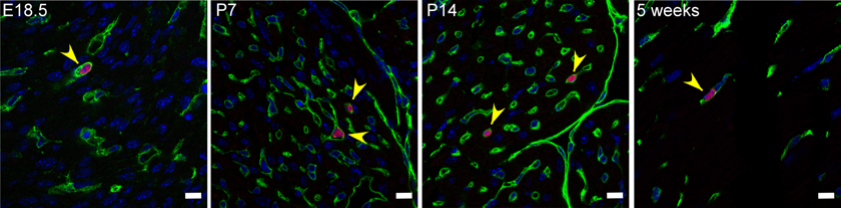
Myocardial-associated B cells are mostly intravascular throughout late embryonic life and early adulthood. Representative immunostained cryosections from the heart of CD19 reporter mice. CD19-tdTomato B cells are red, CD31^+^ endothelial cells green, and nuclear staining (DAPI) is blue. Heart samples were collected at E18.5, P7, P14, and 5 weeks of age. The majority of B cells was intravascular. Pictures of the heart were taken at the level of the papillary muscles (magnification 63×). Scale bars: 10 μm. Yellow arrowheads mark the location of B cells.

We have shown previously that myocardial-associated B cells in the adult heart were primarily intravascular in location and in close approximation with vascular endothelial cells (13). To determine whether B cells were intravascular throughout development, we harvested hearts from CD19-Cre tdTomato reporter mice (13) from E18 to 5 weeks of age. Hearts were fixed and sectioned, and immunofluorescence staining was performed for the endothelial marker CD31. Figure 3 and Supplemental Videos 1–4 show that, in all analyzed time points, the vast majority of myocardial B cells were intravascular and in close association with the endothelium.

### Global changes in B cells during growth and tissue-specific features.

**Figure 4:**
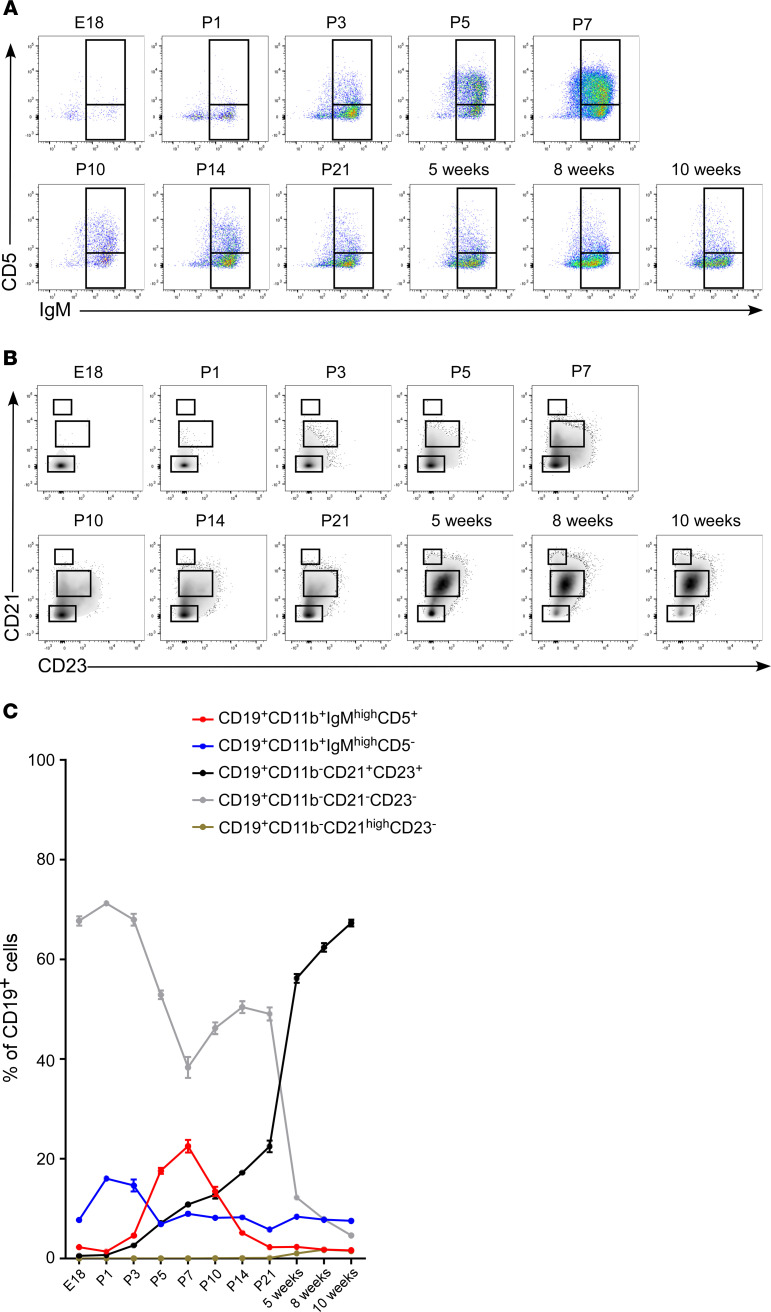
Splenic B cell subsets change from late embryonic life to adulthood. (**A**) Representative flow charts showing the expression of CD5 and IgM in CD19^+^CD11b^+^ splenic B cells from E18 to adult stage (10 weeks old). The box inserts within the charts delineate the IgM^hi^CD5^+^ and IgM^hi^CD5^–^ subsets. The prevalence of IgM^hi^CD5^+^ B cells increases during neonatal life (peak at P7), before decreasing as mice mature toward adulthood. (**B**) Representative flow charts of CD21 and CD23 expression in splenic CD19^+^CD11b^–^ B cells. The box inserts within each chart delineate the CD21^–^CD23^–^, CD21^+^CD23^+^, and CD21^hi^CD23^–^ subsets. (**C**) Summary graph showing the dynamic changes in B cell composition from embryonic to adult life in the spleen. Changes in splenic B cell subsets mirror changes in myocardial B cell subsets. Supplemental Table 3 shows the statistical analysis of each subset from embryonic through adult life. *n* = 3–6 samples/condition. From E18 to P7, 3–6 embryonic and neonatal spleens were pooled together to constitute *n* = 1.

In our prior work, we showed that, in adult mice, myocardial B cells recirculate between the heart and spleen (13). We sought to determine whether the observed changes in cardiac B cell characteristics from the embryo to the adult mirrored changes in splenic B cells from the embryo to the adult. Figure 4, A–C, and Supplemental Table 3 show that the dynamic changes in B cell subsets observed in the heart paralleled changes in B cell subsets occurring in the spleen. For example, in neonatal stages P1–P14, the spleen had a similar increase in the prevalence of CD19^+^CD11b^+^IgM^hi^CD5^+^ cells that we had observed in the heart in the first weeks of postnatal growth. Analysis of spleen B lymphocytes revealed a similar age-related decrease in the pool of CD19^+^CD11b^+^CD21^–^CD23^–^ cells that occurred as the pool CD19^+^CD11b^+^CD21^+^CD23^+^ cells increased, mirroring the changes observed in the neonatal and adult heart (Figure 4, A–C and Supplemental Table 3).

**Figure 5:**
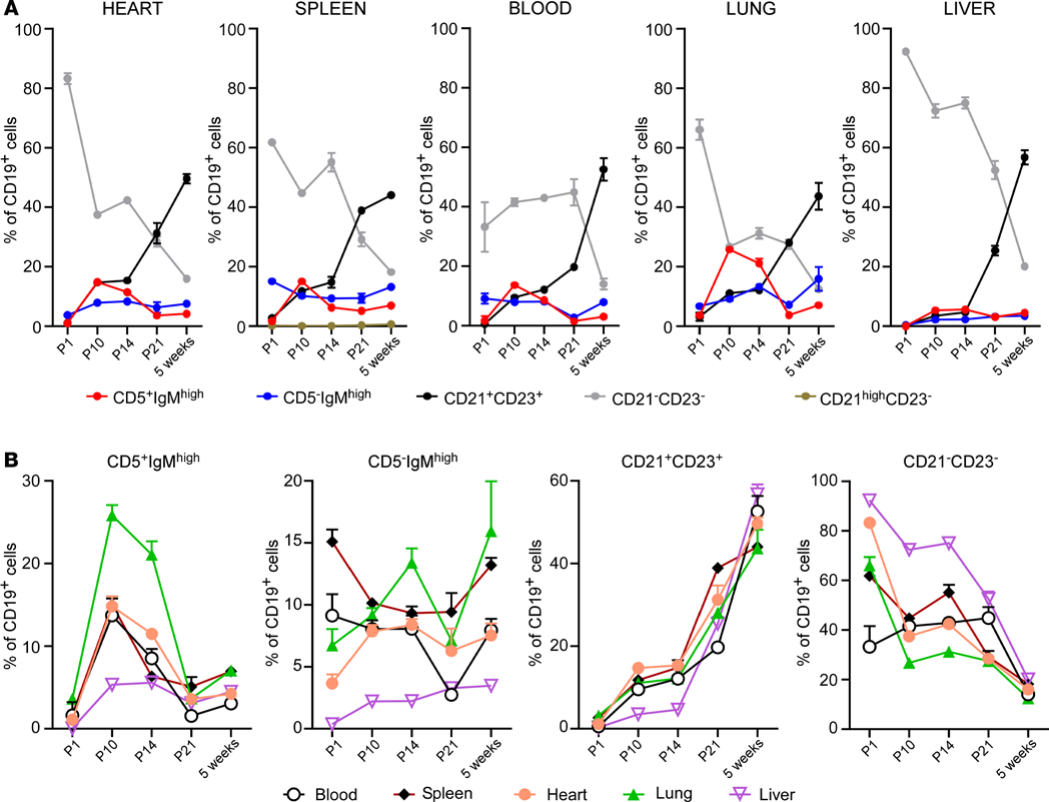
Changes in myocardial-associated B cell subsets mirror changes in B cell subsets observed in spleen, blood, lung, and liver, despite the presence of organ-specific features. (**A**) Group data showing the composition B cell in the heart, spleen, blood, lung, and liver from P1 to 5 weeks of age. B cell composition is similar in all tissues and goes through the same dynamic changes during growth. The IgM^hi^CD5^+^ subset expands in early neonatal life, and the CD21^–^CD23^–^ subset gradually decreases during development as the prevalence of the CD21^+^CD23^+^ subset increases. (**B**) Summary data showing the contribution of each B cell subset across tissues at different time points during growth. Despite marked similarities, tissue-specific features are present at various time points. Supplemental Table 4 shows the statistical analysis of each tissue and subset. *n* = 4–6 samples/condition. At P1, tissues from 3–4 animals were pooled together to constitute *n* = 1.

Given that the dynamic changes in B cell subsets observed in the heart reflected changes observed in the spleen, we next asked whether this relationship was specific for the heart and spleen, or whether it reflected, more broadly, a similar relationship between splenic B cells and B cells in peripheral organs. To address this question, we simultaneously analyzed B cell subsets in the heart, spleen, blood, lung, and liver of neonatal and adult mice. All animals were carefully perfused with HBSS (with Ca^2+^ and Mg^2+^) after blood collection and before organ collection, in order to minimize the possible contamination of peripheral blood B cells in the various organs (for details, see Methods). We found that the dynamic changes in B cell subsets observed in the heart and spleen were also observed in other peripheral organs (Figure 5A). This statement notwithstanding, we noticed that there were several tissue-specific features with respect to B cell ontogeny. As one example, at P14, the prevalence of CD19^+^CD11b^+^IgM^hi^CD5^–^ cells was highest in the lung, intermediate in heart, spleen, and blood, and lowest in the liver (Figure 5B and Supplemental Table 4). In contrast, the prevalence of CD19^+^CD11b^+^CD21^–^CD23^–^ cells in the heart at birth (P1) was significantly different from that observed in blood, lung, and spleen (Figure 5B and *P* value in Supplemental Table 4). Many other tissue-specific differences were identified and are reported in Supplemental Table 4. Viewed together, these data show that the prevalence of B cell subsets changes dynamically in the heart, spleen, blood, lung, and liver during early postnatal life and that, while there is considerable overlap of the B cell subsets in different organs, there are tissue-specific features with respect to tissue-associated B cell subsets in the neonate and adult mouse.

### Myocardial B cells represent a subset of circulating B cells that are in close contact with the microvascular endothelium of the heart and have a unique transcriptional profile.

**Figure 6:**
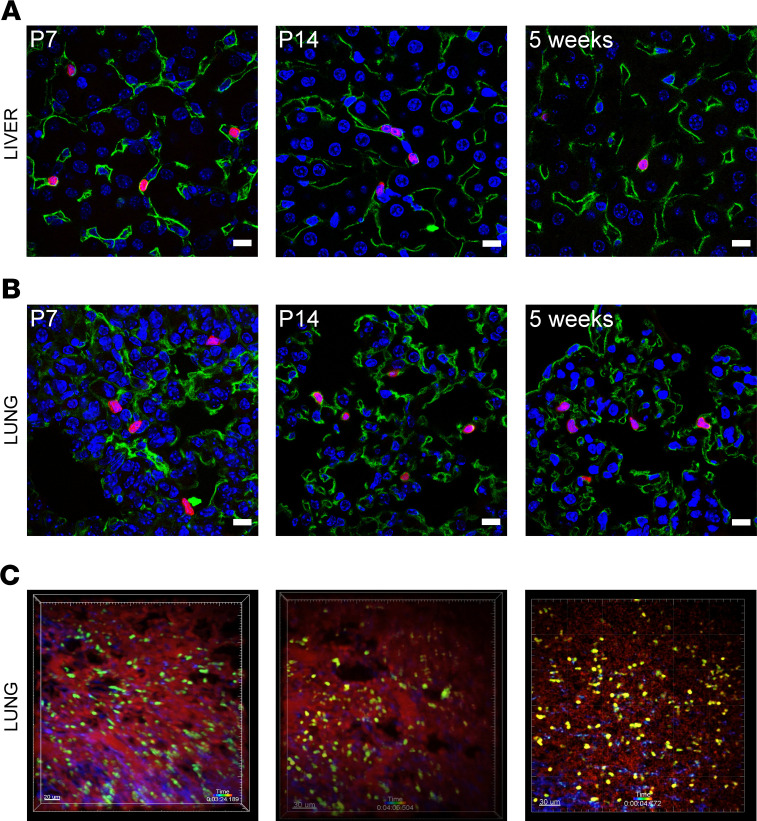
Liver- and lung-associated B cells are mostly intravascular from neonatal to adult life. (**A** and **B**) Representative confocal pictures from neonatal and adult samples, showing that the majority of liver (**A**) and lung (**B**) B cells are intravascular and in association with the endothelium. CD19-tdTomato, red; CD31, green; DAPI, blue. Scale bars: 10 μm. (**C**) Still images from intravital microscopy of B cells flowing through the lung of adult mice. In the lung, B cells were found in the intravascular space, circulating or pausing within the microvasculature. B cells, green; vasculature: red. Scale bars: 30 μm.

**Figure 7:**
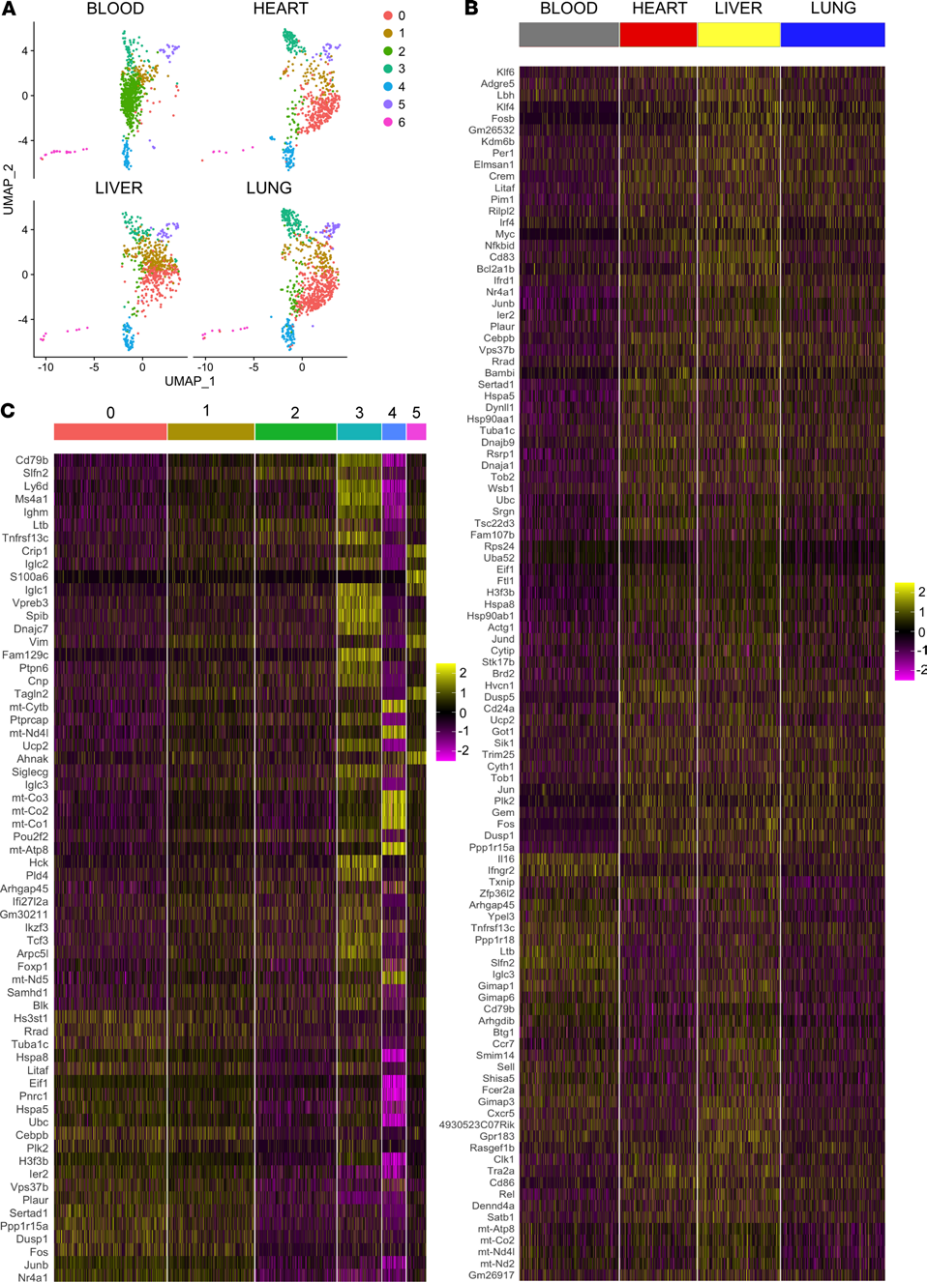
Myocardial-associated B cells are part of a pool of organ-associated B cells transcriptionally distinct from circulating B cells. (**A**) UMAP plot of CD19^+^ cells sorted from the blood and from perfused heart, liver, and lung. B cells from different tissues were collected from the same mice. Unsupervised clustering analysis reveals that all identified clusters were present in all tissues analyzed. However, the prevalence of specific clusters varied. Cluster 0 is abundant in heart, liver, and lung and almost absent from circulating blood. Cluster 2 is highly prevalent in the blood and is rare in tissues. (**B**) Heatmap of the differentially expressed genes in blood, heart, liver, and lung. Transcriptional profile of blood cells is distinct from that of organ-associated B cells. (**C**) Heatmap of the differentially expressed genes in cluster 0. B cells in cluster 0 present a characteristic transcriptional signature when compared with the other clusters.

We have shown previously through histological analysis, intravital microscopy, and 10× single cell sequencing that intravascular myocardial B cells are distinct from circulating B cells and have a unique transcriptional profile (13). The findings in the present study with respect to the concurrent changes in B cell surface markers in the heart and spleen raised the interesting possibility that the subpopulations of B cells identified in the heart might not be unique to the heart but rather might reflect changes in subpopulations of B cells in close approximation with the microvasculature in different organs. To test this possibility, we performed histological analysis of lung and liver, intravital microscopy of the lung, and 10× single cells sequencing of B cells from the heart, blood, lung, and the liver. We first investigated the location of B lymphocytes histologically, by harvesting the lung and liver of CD19-tdTomato reporter mice, and then performing immunofluorescence staining with an anti-CD31 antibody. Immunofluorescence analysis of the liver (Figure 6A and Supplemental Videos 5–7) and lung (Figure 6B and Supplemental Videos 8–10) at P7, P14, and 5 weeks of age showed that the majority of B cells in these tissues were intravascular and in close relationship with the endothelium in neonates and adults, consistent with our previous findings in myocardial tissue (13). We next performed intravital microscopy of the lung of CD19-tdTomato reporter mice (Figure 6C and Supplemental Videos 11–13). We found that lung-associated B cells were predominately intravascular. Some B cells flowed rapidly through the vessels, while other B cells transited more slowly, and some paused on the endothelium, consistent with our prior intravital microscopy studies of myocardial B cells (13). Finally, we performed single cell sequencing of FACS-sorted B cells isolated from blood and perfused heart, liver, and lung. Samples were collected from the same mice, at the same time, and underwent the same digestion and staining protocols. To identify the tissue origin of the B cells, samples were stained with Hashtag antibodies (Supplemental Table 10), processed within the same 10× cDNA library, and sequenced together to eliminate potential “batch” artifacts. Figure 7A shows unsupervised clustering of scRNAseq data from this experiment. We identified the same clusters of B cells in blood, heart, liver, and lung. However, the relative density of specific clusters varied markedly between blood and organs (heart, liver, and lung), whereas the cluster density was similar in heart, liver, and lung. For instance, cluster 0 was abundantly represented in organ-associated (heart, liver, and lung) B cells but not in circulating B cells. Cluster 2 was the largest cluster in B cells from the blood but was rare among organ-associated B cells (Figure 7A). These data suggest that circulating blood B cells have a transcriptional profile that is distinct from that of intravascular B cells associated with liver, lung, and heart, and suggests that myocardial-associated B cells have a transcriptional profile that is similar to that of tissue-associated B cells present in other organs.

To confirm these findings, we first examined the expression of B cell–specific markers (Cd79a, Cd19, and Cd79b) in the above analysis and eliminated any cells that were negative for these markers from further analysis (Supplemental Figure 4). Then, we comparatively analyzed the gene expression profile of B cells from different compartments via heatmaps. Figure 7B shows a heatmap of the differentially expressed genes in B cells sorted from blood, heart, liver, and lung (Supplemental Table 5). These analyses confirmed that circulating B cells have a transcriptional profile distinct from that of organ-associated B cells and show that organ-associated B cells isolated from heart, lung, and liver have remarkable similarities. We explored the functional significance of the gene expression differences between blood and tissue-associated B cells using Gene Ontology (GO) Enrichment Analysis (Table 1) and Kyoto Encyclopedia of Genes and Genomes (KEGG; https://www.genome.jp/kegg) pathways analysis (Supplemental Table 6). When compared with blood B cells, tissue-associated B cells were characterized by a differential regulation of several GO Biological Process Pathways, including cell activation and regulation of immune system process (Table 1), as well as KEGG signaling pathways associated with B cell receptor signaling and antigen processing and presentation (Supplemental Table 6).

Finally, we performed heatmap-based analysis and functional annotation of the genes differentially expressed in cluster zero, which is the cluster most represented within organ-associated B cells. Figure 7C shows that cluster 0 had a transcriptional profile clearly distinct from that of the other 6 clusters (Supplemental Table 7). Table 2 and Supplemental Table 8 show that cells in cluster zero were characterized by GO Biological Process Pathways related to B cell activation and metabolic activation (Table 2) and KEGG signaling pathways (Supplemental Table 8) associated with B cell receptor signaling and oxidative phosphorylation. These findings support the point of view that myocardial-associated B cells are a subset of a larger pool of intravascular organ-associated B cells that are transcriptionally distinct from circulating blood B cells and are characterized by activation of transcriptional pathways consistent with cell activation, increased metabolism, B cell receptor signaling, and possibly antigen processing and presentation.

## Discussion

In the present study, we systematically investigated B cell characteristics in the embryonic, neonatal, and adult mouse heart. We found that B cells are present in the naive heart as early as E13.5 (Figure 1). Consistent with our prior observations in the adult heart (13), we found that naive B cells in the heart are predominantly intravascular throughout embryonic and neonatal life (Figure 1, Figure 2, and Figure 3) and that myocardial B cells are in dynamic equilibrium with splenic B cells (Figure 4 and Figure 5). Surprisingly, we found that myocardial-associated B cells are representative of a much larger, previously unappreciated population of tissue-associated B cells (Figure 6 and Figure 7) that are also in equilibrium with splenic B cells. These findings broaden our understanding of myocardial B cell biology and challenge the current model of naive B cell behavior, which posits that naive B cells continuously recirculate between primary and secondary lymphoid organs without accumulating in peripheral tissue unless there is tissue injury (10–12).

The myocardial leukocyte pool changes during development of the heart, especially within the monocyte/macrophage compartment (15–17). Our findings with respect to the dynamic changes of myocardial-associated B cells during embryonic and early postnatal development (Figure 1) expand upon these observations. By analyzing the expression of CD19, CD11b, CD5, CD21, and CD23, we identified 4 distinct B cell subgroups within the naive murine heart, whose relative expression changed markedly throughout development, including CD19^+^CD11b^+^CD5^+^, CD19^+^CD11b^+^CD5^–^, CD19^+^CD11b^–^CD21^+^CD23^+^, and CD19^+^CD11b^–^CD21^–^CD23^–^ B cells (Figure 1). The population of CD19^+^CD11b^+^ cells were IgM^hi^, which is a classic signature of B1 cells (18). Within this subgroup, CD5 expression discriminates B1a cells (CD5^+^, only of embryonic origin) from B1b cells (CD5^–^, both of embryonic origin and of BM origin) (18, 19). In contrast, CD19^+^CD11b^–^ cells are strictly BM-derived (B2) cells (18). Our data show that the prevalence of CD19^+^CD11b^+^ B1 cells decreases in concert with a reciprocal increase in the prevalence of CD19^+^CD11b^–^ B2 cells during the transition from embryonic to adult life (Figure 1), consistent with the embryonic origin of B1 cells. Since B2 cells are typically BM derived, these findings suggests that embryonic B cells do not reside within the parenchyma, as we have reported for embryonic macrophages (16, 20), which are self-replicating and remain resident within the parenchyma of the myocardium throughout adult life. Fate mapping studies of CD19^+^CD11b^–^ and CD19^+^CD11b^+^ myocardial-associated B cells will be needed to confirm these findings.

To further refine our identification of B cell subsets based on surface markers, we performed 10× scRNAseq. Gene expression analysis of the Immgen RNAseq data base (Immunological Genome Project) indicated that the CD11b^+^ cells were B1 cells (Figure 2F). scRNAseq analysis of CD11b^–^ cells revealed that the murine heart harbors mainly 2 subpopulations of B2 cells. A population of CD21^–^CD23^–^ cells was detected as early as E15.5, it peaked on P1, and was found to be comprised of early transitional B cells (Figure 2D). A population of CD21^+^CD23^+^ cells was detectable as early as P5, peaked in the adult heart, and was found to be comprised mostly of naive, T3/FO cells (Figure 2E). These findings are consistent with the observation that transitional B cells are immature B cells that migrate from the BM into the spleen (21), whereas T3/FO B cells are recirculating B cells that mature in the spleen (22). These data confirm our earlier observations that the majority of myocardial B cells in the murine heart are naive follicular B cells (13). Our pseudotime analysis showed that the trajectory of CD21^–^CD23^–^ cells moved in direction to CD21^+^CD23^+^ cells (Figure 2C), further suggesting that CD21^–^CD23^–^ cells represent immature B cells that are destined to become CD21^+^CD23^+^ B cells. Although pseudotime estimation is not a definitive means for determining cell fate, at the time of this writing, we are unaware of specific cell fate markers for B cells that would allow a more definitive analysis. Accordingly, the studies of pseudotime estimation should be regarded as provisional.

We have shown previously that B cells in the adult heart are predominately intravascular and are part of a larger population of B cells that circulates between the heart, blood, spleen, and other organs (13). The observation in the present study that the age-related changes in the composition of myocardial-associated B cells are mirrored by age-related changes in splenic B cells are in agreement with our previous findings and strongly suggest that B cells circulate between the heart, the blood, and the spleen in the embryo, neonatal, and adult mouse (Figure 4). These observations prompted us to ask whether there might be similar age-related changes in B cell populations in other tissue beds. To test this hypothesis, we first performed a flow cytometric analysis of temporal changes in B cell populations in the blood, liver, lung, spleen, and heart (Figure 5). This analysis revealed that the age-related temporal changes in B cell subsets observed in one tissue mirrored those observed in all the other tissues that were examined (Figure 5). Immunofluorescence studies showed that, analogous to the heart, B cells in the lung and liver were almost exclusively intravascular and were in close approximation to endothelial cells (Figure 6 and Supplemental Videos 5–10). Intravital microscopy of the lung revealed that circulating B cells paused in the lung microvasculature, mimicking a behavior that we had previously observed performing intravital microscopy on the heart (Figure 6 and Supplemental Videos 11–13; ref. 13).

To further explore the relationship between circulating B cells and B cells in the heart, lung, and liver, we simultaneously collected blood and tissue samples and performed 10× scRNAseq on each of these samples. Clustering analysis revealed that there were 7 unique B cell clusters in the blood, heart, liver, and lung; however, the number of cells within each cluster differed depending on whether the B cells were freely circulating in the blood or whether the B cells were associated to tissue beds. As shown, the relative numbers of cells within clusters 0–6 were similar in the heart, lung, and liver, whereas the number of cells in cluster 2 from blood B cells was increased relative to the number of cells in cluster 2 in the heart, lung, and liver (Figure 7A). Pathway analysis of the genes that were differentially expressed in B cells in tissue vs. blood (Table 1 and Supplemental Table 6) revealed that B cells associated to the heart, liver, and lung had enrichment of gene pathways associated with increased metabolic activity, active B cell receptor signaling, and active antigen presentation pathways (Table 1 and Supplemental Table 6). The cluster analysis also identified a specific B cell cluster that was virtually absent from the blood but was enriched in the heart, liver, and lung (cluster 0; Figure 7A). Functional pathway analysis of genes differentially expressed within this cluster (Figure 7C) revealed that this cluster was enriched for genes that were associated with B cell activation, B cell receptor signaling, increased metabolic activity, and oxidative phosphorylation receptor (Table 2 and Supplemental Table 8). Although this analysis does not address the important question of whether there are populations of circulating B cells that become activated in order to adhere to vascular endothelial cells, or whether the circulating B cells randomly attach to the vascular endothelium where they become activated, the observation that B cells in cluster 0 were enriched within tissues but were virtually nonexistent in the blood suggests that circulating B cells become activated once they come into close proximity to vascular endothelial cells, where they may play a role in immune surveillance of membrane-bound antigens present on the endothelial surface. Consistent with this point of view, our functional pathway analysis shows that the population of intravascular B cells associated with tissues is characterized by activity within the B cell receptor signaling and antigen presentation pathways. Further studies will be needed to define the biological mechanism that underscores these transcriptional differences.

In summary, in the present report, we have characterized the dynamic age-related changes in naive myocardial B1 and B2 cells in the embryonic, neonatal, and the adult heart. Our findings show that the changes in subsets of intravascular myocardial B cells are mirrored by changes in splenic B cells, suggesting that B cells circulate between the heart and spleen throughout life. Moreover, and to some extent surprisingly, our studies suggest that the temporal changes in subsets of intravascular myocardial B cells are not unique to the heart, but rather are broadly reflective of changes in intravascular B cells located in other organs, such as the liver and the lung. These findings indicate that current models of the dynamics of the naive B cells are incomplete. Germane to this discussion, both our flow cytometric analysis and our gene expression analysis highlighted that, despite having many overlapping phenotypic and transcriptional profiles, the populations of B cells pools associated with different organs have unique tissue-specific features (Figure 5B, Figure 7, A and B, and Supplemental Tables 4 and 5). The biological significance, if any, of these tissue-specific characteristics remains unclear at present; accordingly, further work will be needed to investigate the functional significance of organ-associated B cells, both at baseline and in the context of tissue injury.

## Methods

### Mice

To study B cell subsets during development, we used male and female C57BL/6J (stock no. 000664) mice between embryonic age E13.5 and early adulthood (10 weeks old). These mice, including timed-pregnant females, were purchased from The Jackson Laboratory. To generate a CD19 reporter model, CD19-Cre male mice (C57BL/6J; B6.129P2(C)-*Cd19^tm1(cre)Cgn^*/J; stock no. 006785) were bred with Rosa-tdTomato females (C57BL/6J; B6.Cg-*Gt(ROSA)26Sor^tm14(CAG–TdTomato)Hze^*/J; stock no. 007914), as previously described (13). The F1 generation was employed for all the experiments. Mice were bred and maintained at the Washington University School of Medicine.

### Flow cytometric analysis

#### Isolation of B cells from digested tissues.

For flow cytometry experiments, mice were euthanized by either decapitation (when younger than 7 days old) or CO_2_ inhalation. Hearts were perfused with ~5 mL of cold HBSS with Ca^2+^ and Mg^2+^. Any extracardiac tissue was removed, and hearts were finely minced. In preliminary experiments, we determined that tissue digestion using Collagenase type I (MilliporeSigma; catalog C0130) affected the expression of the B cell surface markers CD21 and CD23, whereas the use of purified Collagenase (Worthington; catalog LS005277) resulted in the preservation of these molecules (Supplemental Figure 1). Accordingly, hearts were digested with purified Collagenase (Worthington; catalog LS005277; 200 U/mL), DNAse I type II (MilliporeSigma; catalog D4527; 60 U/mL), and hyaluronidase (MilliporeSigma; catalog H3506; 60 U/mL) in a final volume of 3 mL of HBSS for 30 minutes at 37°C with agitation. A total of 6 mL of a 2% FBS/2% BSA solution in HBSS was added to block the enzymatic activity. The digested sample was filtered using 40 μm cell strainers and centrifuged at 400 *g* for 7 minutes at 4°C. RBCs were lysed using ACK Lysis Buffer (Thermo Fisher Scientific; catalog A10492). The remaining cells were resuspended in 200 μL of FACS Buffer (2% FBS, 2 μM EDTA in sterile PBS) and stained with conjugated antibodies (Supplemental Table 6) for 30–45 minutes at 4°C and washed with FACS Buffer before analysis. Embryonic hearts were dissected under a binocular microscope and pooled in groups of 3–5 hearts per tube for downstream analysis. Digestion of the embryonic tissue was performed in 1 mL of HBSS containing purified Collagenase (Worthington; catalog LS005277; 200 U/mL), DNAse I type II (MilliporeSigma; catalog D4527; 60 U/mL), and hyaluronidase (MilliporeSigma; catalog H3506; 60 U/mL) for 30 minutes at 37°C with agitation. Supplemental Table 9 shows the antibodies used in this study. Compensation controls were generated using UltraComp ebeads (Invitrogen; catalog 01-2222-42). Positive cells were determined by the use of single-color control samples from heart and spleen. FMO staining was used as an additional staining control. Dead cells were excluded using the Zombie Aqua dye (BioLegend; catalog 423102). Data were acquired using BD LSR-Fortessa X20 analyzer at the Washington University Department of Pathology Flow Cytometry and Sorting Core facility. The gating strategy used is shown in Supplemental Figure 2 (heart).

#### Collection of blood, heart, spleen, liver, and lung B cells.

Trunk blood was obtained after euthanasia and collected into tubes containing heparin. Prior to collection of heart, liver, and lung, animals were perfused as follows. The abdominal and thoracic cavities were exposed. The left kidney was removed to create an exit point for blood and perfusing solution. A 27–20 G needle connected with a perfusion system containing HBSS (with Ca^2+^ and Mg^2+^) was inserted into the right ventricle. Animals were perfused with 8–10 mL of HBSS. Tissue coloration was observed, and only tissues that became pale through perfusion (as they were cleared of intravascular blood) were used for further analysis. Perfused organs were removed and transferred to a tube containing cold HBSS. Spleens were removed and finely minced using 40 μm filters in HBSS. Blood, heart, spleen, liver, and lung were collected from the same mice and digested as described in the “Isolation of B cells from digested tissues” section.

### Cell sorting and single cell transcriptional profiling

We used the Chromium Single Cell 3′ v3 or 5′ Library Kit and Chromium instrument (both from 10x Genomics) to perform single cell transcriptional profiling analyses.

#### Neonatal and adult myocardial B cells.

We performed transcriptional profiling of cardiac B cells isolated from 6 neonatal (2 weeks old) and 6 adult (8 weeks old) WT mice. In this experiment, we did 3′ sequencing. B cells were isolated as described in the “Isolation of B cells from digested tissues” section. Neonatal and adult samples were treated in exactly the same way. After removing RBCs, the remaining cells were resuspended in 200 μL of FACS Buffer containing 1% of TruStain FcX Plus (BioLegend; catalog 156604; Fc Blocker). A total of 2 μL of Zombie Aqua Fixable Viability Kit (BioLegend; catalog 423102) was added to each sample. Neonatal samples were pooled in 2 tubes with 3 hearts/tube. Adult samples were also pooled in 2 tubes with 3 hearts/tube. A total of 1 μL of CD45/PerCP-Cy5 and CD19/APC antibody (Supplemental Table 9) was added to each tube. A total of 4 μL of CD21, CD23, and CD11b Oligo-Antibody (Supplemental Table 10 for details) was added to each tube. Cells were incubated for 30 minutes at 4°C protected from light and then washed twice with FACS Buffer. Twenty-seven thousand CD45^+^Aqua^–^ CD19^+^ cells (doublets excluded) were sorted in each group using a FACS Aria-II Cell Sorter. Sorted B cells were collected in PBS (0.4% BSA). CD45^+^Aqua^–^CD19^+^ sorted cells were centrifuged at 1000 *g*, 4°C for 10 minutes (acceleration 7 and break 0) and resuspended in 20 μL of PBS 0.4% BSA. The neonatal and adult samples were submitted to the MacDonnell Genome Institute at Washington University for further processing. Sample processing and analyses were performed as previously described (13). Neonatal and adult B cells were digested and sorted following the same experimental conditions and on the same day. B cell identity in CD21^+^CD23^+^, CD21^–^CD23^–^, and CD11b^+^ groups was determined by comparing the upregulated genes in each subset to known RNAseq databases provided by the Immunological Genome Project (Immgen, http://rstats.immgen.org/MyGeneSet_New/index.html). The data are available on NCIB GEO repository accession number GSE153568 (https://www.ncbi.nlm.nih.gov/geo/query/acc.cgi?acc=GSE153568).

#### Heart, blood, lung, and liver.

To compare the gene expression profile of heart, blood, lung, and liver, we used adult WT mice at 10 weeks of age. In this experiment, we performed 5′ sequencing. Heart, blood, lung, and liver were isolated from the same mice. Digestion and staining protocols were described in “Neonatal and Adult B cells”. We collected 5000 blood B cells, 5000 lung B cells, 5000 liver B cells, and 5000 heart B cells. They were all labeled with Hashtag-C antibodies. Blood was labeled with Hashtag-C #1, lung with Hashtag-C #2, liver with Hashtag-C #3, and heart with Hashtag-C #4 (See Supplemental Table 10 for antibody details). Blood, heart, lung, and liver were submitted to the same digestion protocol, and B cells were sorted on the same day. Gene expression analysis was performed only in cells that were positive for B cell markers (see Supplemental Figure 4 for details). Pathway analysis of genes differentially expressed in blood, heart, lung, and liver B cells was performed using the GSEA software (MSigDB version 7.0) (23, 24). GO Enrichment Analysis and KEGG pathway analysis were used. Only pathways related to signal transduction, immune system, and energy metabolism were used in KEGG pathway analysis. The data are available on NCIB GEO repository accession number GSE153569 (https://www.ncbi.nlm.nih.gov/geo/query/acc.cgi?acc=GSE153569).

### scRNAseq bioinformatics

Single cell expression data were processed using Cell Ranger Single Cell Software Suite v3.1.0 for sample demultiplexing, alignment, filtering, UMI counting, and data aggregation (https://support.10xgenomics.com/single-cell-gene-expression/software/pipelines/latest/what-is-cell-ranger). Differential expression analyses were preformed using R package Seurat (25). Pseudotime analyses were preformed using R Package Monocle 3 (26).

### CD31 staining in the heart, lung, and liver of CD19 reporter mice

Hearts, lung, and liver from CD19 reporter mice were perfused with HBSS as described in “Collection of blood, heart, spleen, liver, and lung B cells”. Tissues were fixed in 3.7% formalin for 24 hours at 4°C and then transferred to 30% sucrose for 24 hours before embedding in OCT and flash freezing. Sections (10 μm) were blocked with a solution 2% FCS and 2% blocking reagent (Roche; catalog 11 096 176 001) in maleate buffer (100 mM Maleic Acid, 150 mM NaCl, pH 7.5) for 1 hour at room temperature. Sections were stained with the primary antibody for anti-CD31 (R&D Systems; catalog AF3628; 1:15), overnight at 4°C, followed by incubation with NL637 secondary antibody (R&D Systems; catalog NL002; 1:200) for 4 hours at room temperature. DAPI was used as a nuclear counterstain. Slides were imaged using a Zeiss LSM 880 Confocal microscope (63× magnification for objective lenses) at the Washington University Center for Cellular Imaging (WUCCI). Pictures of the heart were taken at the level of the papillary muscles (63× magnification for objective lenses).

### Intravital microscopy

Intravital imaging of the lung of adult B6 CD19-tdTomato mice was performed using a custom-built 2-photon microscope running ImageWarp version 2.1 software (A&B Software) as described previously (13). Time-lapse imaging of lymphocyte trafficking in the lung was performed by averaging 15 video-rate frames (0.5 seconds per slice) captured during the acquisition to match the ventilator rate and minimize movement artifacts. Each plane represents an image measuring 220–240 μm in the *x* and *y* dimensions. Twenty-one sequential planes were acquired in the *z* dimension (2.5 μm each) and compiled to generate a *Z*-stack. Blood vessels were visualized by i.v. injection of 50 μL of PBS containing 20 μL of 655-nm nontargeted Q-dots.

### Statistics

All results are presented as mean ± SEM. Statistical comparisons were performed using 2-way ANOVA followed by Tukey’s post hoc test. Data were analyzed with GraphPad Prism Version 8.

### Study approval

All studies were performed with the approval of the IACUC at Washington University School of Medicine. These investigations conform to the *Guide for the Care and Use of Laboratory Animals* (National Academies Press, 2011). Experiments were performed according to protocols approved by the IACUC at Washington University School of Medicine.

## Author contributions

CRR designed the experiments, performed the experiments, and wrote the manuscript. LA designed the experiments, performed the experiments, supervised the experiments, and wrote the manuscript. DLM supervised the experiments and wrote the manuscript. WY performed single cell transcriptional profiling analysis. WL and DK performed intravital microscopy. All authors approved the final version of the manuscript.

## Supplementary Material

Supplemental data

Supplemental Video 1

Supplemental Video 2

Supplemental Video 3

Supplemental Video 4

Supplemental Video 5

Supplemental Video 6

Supplemental Video 7

Supplemental Video 8

Supplemental Video 9

Supplemental Video 10

Supplemental Video 11

Supplemental Video 12

Supplemental Video 13

## Figures and Tables

**Table 1:**
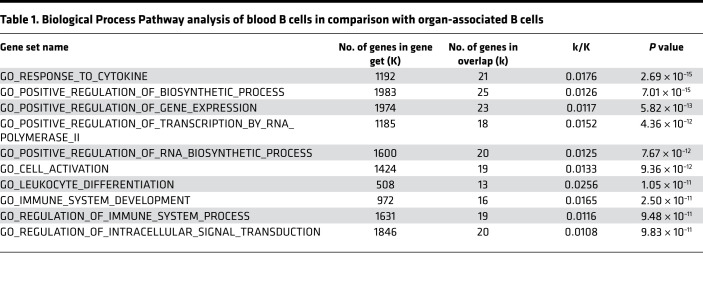
Biological Process Pathway analysis of blood B cells in comparison with organ-associated B cells

**Table 2:**
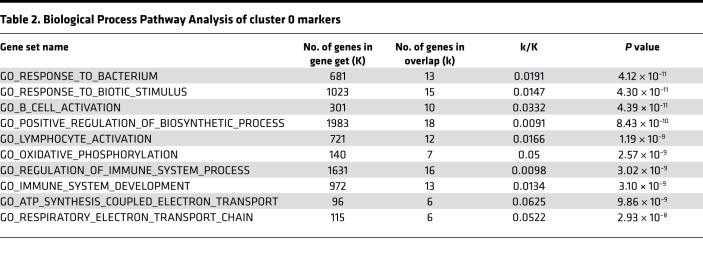
Biological Process Pathway Analysis of cluster 0 markers

## Version 1 07/14/2020

In-Press Preview

## Version 2 08/21/2020

Electronic publication

## Version 3 08/26/2020

Updated Supplemental Videos
