# Supplementary material for: Developmental changes in myocardial B cells mirror changes in B cells associated with different organs
Source: JCI Insight. 2020 Aug 20;5(16):e139377. doi: 10.1172/jci.insight.139377 (PMC7455131; doi:10.1172/jci.insight.139377)
Supplement: Supplemental data [file jciinsight-5-139377-s275.pdf]

Supplemental Figures

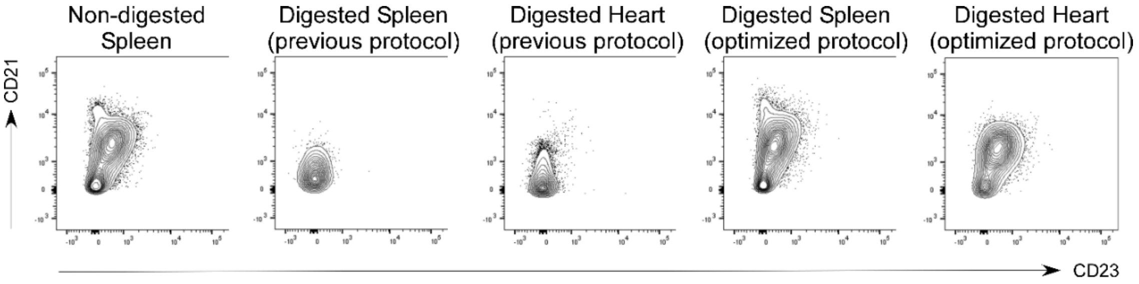

Figure S1: Optimization of tissue digestion protocol to prevent the cleavage of CD21 and CD23. In non-digested spleens, three populations of B cells are distinguished based on the expression of CD21 and CD23: $CD21^{high}CD23^{-}$, $CD21^{+}CD23^{+}$, and $CD21^{-}CD23^{-}$. Digestion with Collagenase using our previously published protocol (1) reduced staining for CD21 and CD23 in B lymphocytes isolated from the spleen and the heart. The use of purified Collagenase (optimized protocol) resulted in the preservation of these antigens in the spleen (positive control) and highlighted expression of CD21 and CD23 in myocardial B cells.

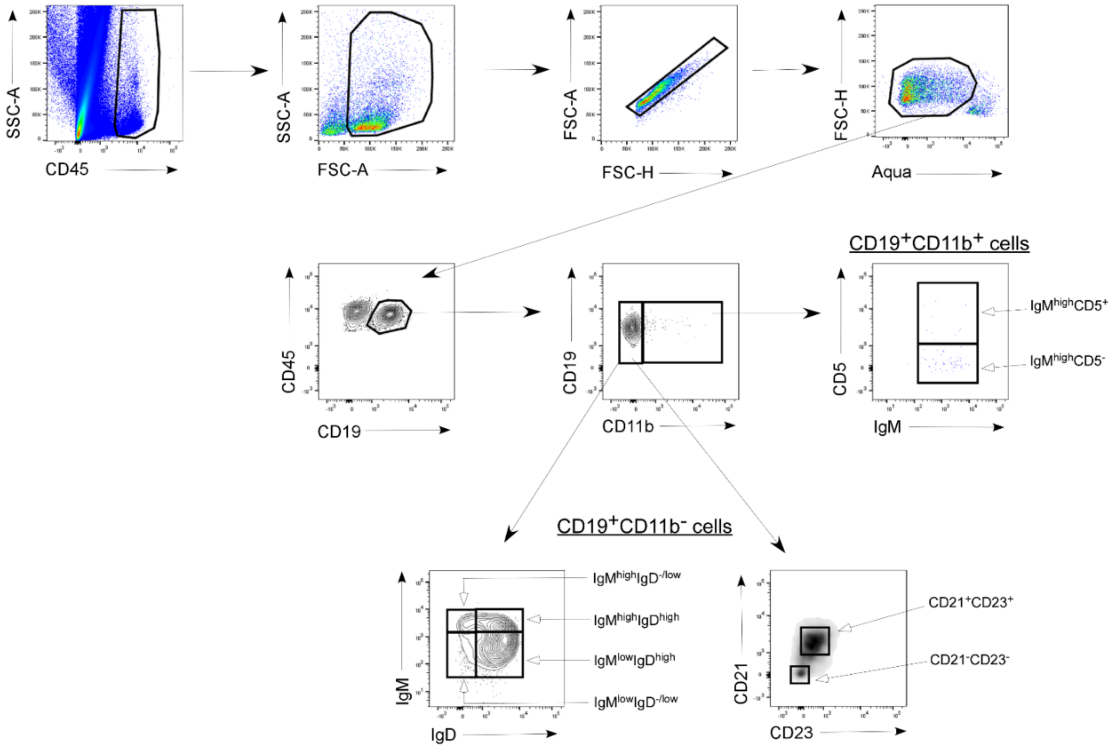

Figure S2: Gating strategy for the flow cytometric analysis of myocardial-associated B cells. B cells were gated as $CD45^{+}Aqua^{-}CD19^{+}$. Most B cells were $CD11b^{-}$ and further analyzed based on the expression of IgM, IgD, CD21, and CD23. $CD11b^{+}$ B cells were analyzed based on the expression of CD5 and IgM.

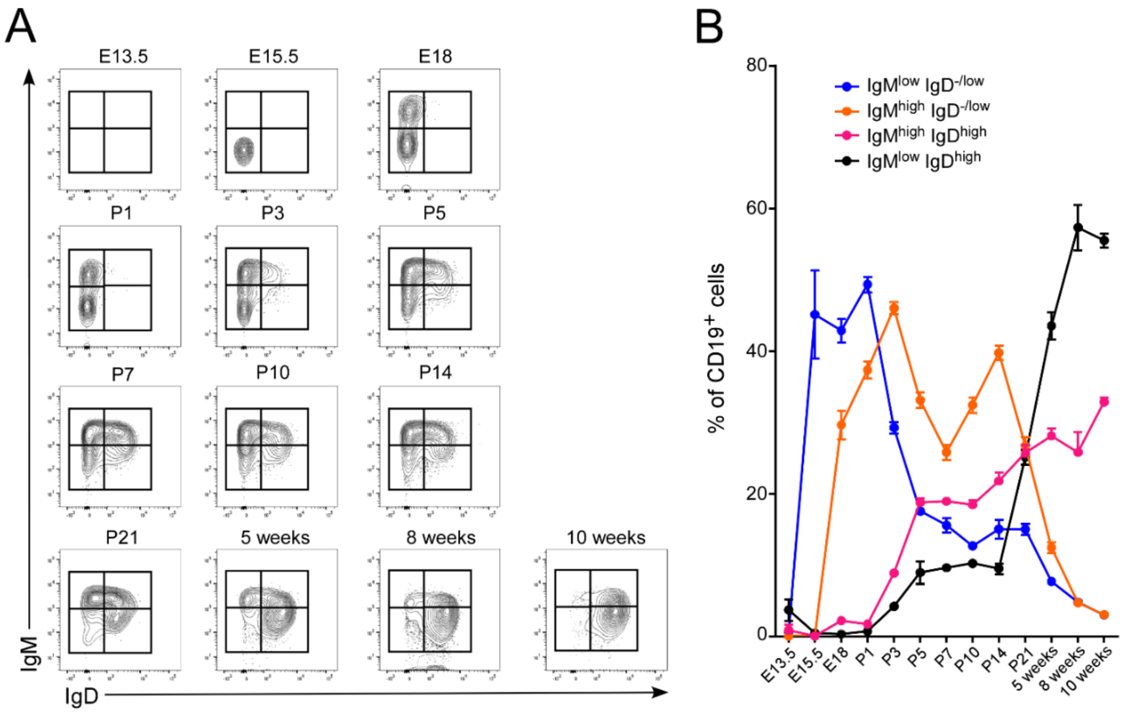

Figure S3: Flow cytometric profile of $CD19^{+}CD11b^{-}$ B cells showing the expression of IgM and IgD from embryonic to adult life. A) Representative flow charts showing the expression of IgM and IgD in $CD19^{+}CD11b^{-}$ B cells from embryonic to adult life in the heart. A population of $IgM^{low}IgD^{-/low}$ cells first appeared in the heart at E15.5. $IgM^{high}IgD^{low}$ cells were seen in the heart at E18. In the early post-natal life, the prevalence of $IgM^{high}IgD^{high}$ cells increased, and the prevalence of $IgM^{low}IgD^{-/low}$ decreased. In the adult heart, the majority of $CD19^{+}CD11b^{-}$ B cells were $IgM^{low}IgD^{high}$. **B)** Group-graph showing the dynamic changes in B cell composition from embryonic to adult life in the heart. n=4-7 samples/condition. From E13.5 to P7, 3-6 embryonic and neonatal hearts were pooled together in the same tube to constitute n=1.

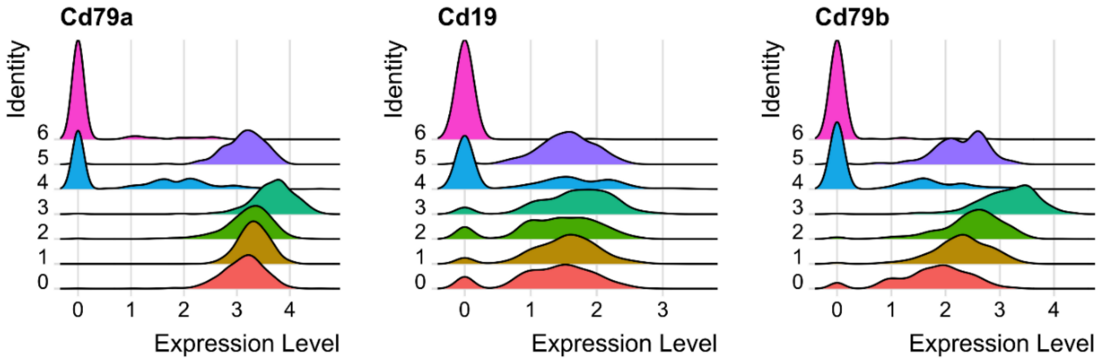

Figure S4: Expression of B cell specific markers in CD45$^+$Aqua$^-$CD19$^+$ B cell clusters. B cells were sorted from blood, heart, liver, and lung. We performed unsupervised clustering of single-cell transcription data and identified 7 clusters (0 to 6). For the subsequent analysis, only cells that were positive for B cell specific markers (Cd79a, Cd19, and Cd79b) were used (cluster 6 and negative cells within cluster 4 were excluded).

Supplemental Table 1: Comparative analysis of B cell subsets ratio in the heart from embryonic day 13.5 (E13.5) to adulthood (10 weeks old). Data were analyzed by 2-way ANOVA followed by Tukey's post-hoc test to correct for multiple comparisons.

	p value			
	**$IgM^{high}CD5^{+}$**	**$IgM^{high}CD5^{-}$**	**$CD21^{+}CD23^{+}$**	**$CD21^{-}CD23^{-}$**
E13.5 vs. E15.5	0.9752	0.8299	n.a.	0.0504
E13.5 vs. E18	0.9914	0.8333	**0.0848**	**<0.0001**
E13.5 vs. P1	0.8916	0.5475	**0.0021**	**<0.0001**
E13.5 vs. P3	0.9900	0.6271	**0.0026**	**<0.0001**
E13.5 vs. P5	0.8948	0.7611	0.1667	**<0.0001**
E13.5 vs. P7	0.3486	0.8406	**<0.0001**	**<0.0001**
E13.5 vs. P10	0.5651	0.7948	**0.0007**	**<0.0001**
E13.5 vs. P14	>0.9999	0.6695	**<0.0001**	**<0.0001**
E13.5 vs. 3 weeks	0.9131	0.4794	**0.0003**	**<0.0001**
E13.5 vs. 5 weeks	0.9192	0.5455	**<0.0001**	**0.0127**
E13.5 vs. 8 weeks	0.8627	0.4267	**<0.0001**	0.3981
E13.5 vs. 10 weeks	0.8737	0.4279	**0.0006**	0.7456
E15.5 vs. E18	0.6658	0.9999	0.0848	0.1389
E15.5 vs. P1	0.5287	0.9998	**0.0021**	0.0537
E15.5 vs. P3	0.6609	>0.9999	**0.0026**	0.0779
E15.5 vs. P5	>0.9999	>0.9999	0.1667	0.9043
E15.5 vs. P7	0.9962	0.9996	**<0.0001**	>0.9999
E15.5 vs. P10	>0.9999	>0.9999	**0.0007**	>0.9999
E15.5 vs. P14	0.8475	>0.9999	**<0.0001**	0.8772
E15.5 vs. 3 weeks	0.5464	0.9936	**0.0003**	>0.9999
E15.5 vs. 5 weeks	0.5510	0.9998	**<0.0001**	0.1415
E15.5 vs. 8 weeks	0.5078	0.9647	**<0.0001**	0.0799
E15.5 vs. 10 weeks	0.5154	0.9666	**0.0006**	0.0715
E18 vs. P1	0.2096	**0.0308**	0.9994	**0.0020**
E18 vs. P3	>0.9999	0.1487	0.0699	**0.0377**
E18 vs. P5	**<0.0001**	0.9646	0.2216	**0.0010**
E18 vs. P7	**<0.0001**	>0.9999	**<0.0001**	**<0.0001**
E18 vs. P10	**0.0037**	0.9999	**0.0005**	**<0.0001**
E18 vs. P14	0.0836	0.3359	**<0.0001**	**<0.0001**
E18 vs. 3 weeks	0.3260	**0.0080**	**0.0002**	**<0.0001**
E18 vs. 5 weeks	0.4651	**0.0287**	**<0.0001**	**<0.0001**
E18 vs. 8 weeks	0.1208	**0.0042**	**<0.0001**	**<0.0001**
E18 vs. 10 weeks	0.1496	**0.0029**	**0.0006**	**<0.0001**
P1 vs. P3	**0.0010**	0.3758	**0.0187**	**0.0098**
P1 vs. P5	**<0.0001**	**0.0046**	0.2113	**0.0002**
P1 vs. P7	**0.0001**	**0.0004**	**<0.0001**	**<0.0001**
P1 vs. P10	**0.0047**	**0.0017**	**0.0008**	**<0.0001**
P1 vs. P14	**<0.0001**	0.0837	**<0.0001**	**<0.0001**
P1 vs. 3 weeks	0.8287	0.5443	**0.0003**	**<0.0001**
P1 vs. 5 weeks	0.9965	>0.9999	**<0.0001**	**<0.0001**
P1 vs. 8 weeks	0.7971	**0.0345**	**<0.0001**	**<0.0001**
P1 vs. 10 weeks	0.9932	0.1039	**0.0007**	**<0.0001**
P3 vs. P5	**<0.0001**	**0.0106**	0.3162	**0.0007**
P3 vs. P7	**0.0002**	**0.0003**	**<0.0001**	**<0.0001**
P3 vs. P10	**0.0075**	**0.0035**	**0.0008**	**<0.0001**
P3 vs. P14	**0.0023**	0.9242	**<0.0001**	**<0.0001**

P3 vs. 3 weeks	**0.0032**	**0.0143**	**0.0003**	**<0.0001**
P3 vs. 5 weeks	0.0770	0.3779	**<0.0001**	**<0.0001**
P3 vs. 8 weeks	**0.0003**	**0.0002**	**<0.0001**	**<0.0001**
P3 vs. 10 weeks	**0.0015**	**0.0096**	**0.0007**	**<0.0001**
P5 vs. P7	**0.0110**	0.1332	0.9999	0.0832
P5 vs. P10	0.4133	0.9244	0.9267	0.1048
P5 vs. P14	**<0.0001**	0.1181	0.2949	>0.9999
P5 vs. 3 weeks	**<0.0001**	**0.0004**	**0.0035**	**0.0439**
P5 vs. 5 weeks	**<0.0001**	**0.0030**	**<0.0001**	**<0.0001**
P5 vs. 8 weeks	**<0.0001**	**<0.0001**	**<0.0001**	**<0.0001**
P5 vs. 10 weeks	**<0.0001**	**0.0019**	**<0.0001**	**0.0001**
P7 vs. P10	0.8852	0.7541	**0.0232**	>0.9999
P7 vs. P14	**0.0002**	**0.0022**	**0.0008**	**0.0307**
P7 vs. 3 weeks	**0.0001**	**<0.0001**	**0.0008**	>0.9999
P7 vs. 5 weeks	**<0.0001**	**0.0002**	**<0.0001**	**<0.0001**
P7 vs. 8 weeks	**<0.0001**	**<0.0001**	**<0.0001**	**<0.0001**
P7 vs. 10 weeks	**<0.0001**	**0.0004**	**0.0009**	**<0.0001**
P10 vs. P14	**0.0158**	**0.0288**	0.0941	**0.0311**
P10 vs. 3 weeks	**0.0051**	**0.0002**	**0.0006**	0.9985
P10 vs. 5 weeks	**0.0036**	**0.0011**	**<0.0001**	**<0.0001**
P10 vs. 8 weeks	**0.0042**	**<0.0001**	**<0.0001**	**<0.0001**
P10 vs. 10 weeks	**0.0041**	**0.0008**	**0.0004**	**<0.0001**
P14 vs. 3 weeks	**0.0001**	**0.0029**	**0.0016**	**0.0034**
P14 vs. 5 weeks	**0.0001**	0.0802	**<0.0001**	**<0.0001**
P14 vs. 8 weeks	**<0.0001**	**<0.0001**	**<0.0001**	**<0.0001**
P14 vs. 10 weeks	**<0.0001**	**0.0032**	**0.0002**	**<0.0001**
3 weeks vs. 5 weeks	>0.9999	0.6136	**<0.0001**	**<0.0001**
3 weeks vs. 8 weeks	0.1508	0.4818	**<0.0001**	**<0.0001**
3 weeks vs. 10 weeks	0.5533	0.7636	**0.0001**	**<0.0001**
5 weeks vs. 8 weeks	0.7906	**0.0348**	**0.0016**	**0.0192**
5 weeks vs. 10 weeks	0.9368	0.1127	0.0738	**0.0075**
8 weeks vs. 10 weeks	>0.9999	>0.9999	0.9670	0.6045

*n.a. = not applicable: Mean Difference = 0.

Supplemental Table 2: List of the unique upregulated genes in CD21⁻CD23⁻, CD21⁺CD23⁺ and CD11b⁺ B cell subsets assessed by seruat.

CD21$^-$CD23$^-$					
**genes**	**p value**	**Avg logFC**	**pct.1**	**pct.2**	**P value adjusted**
Iglc1	1.30E-214	1.397333	0.849	0.517	4.03E-210
Vpreb3	1.97E-94	1.15496	0.619	0.425	6.11E-90
Ighm	0	1.041216	0.974	0.951	0
Ly6d	1.06E-187	0.982473	0.932	0.834	3.28E-183
Ms4a1	9.02E-209	0.982102	0.906	0.784	2.80E-204
Spib	3.74E-169	0.925162	0.716	0.383	1.16E-164
Cd79b	7.85E-243	0.882916	0.94	0.876	2.44E-238
Tagln2	1.85E-84	0.862385	0.611	0.387	5.75E-80
Rilpl2	5.72E-133	0.843463	0.623	0.316	1.78E-128
Iglc2	9.12E-44	0.794945	0.773	0.751	2.83E-39
Pafah1b3	3.19E-137	0.788188	0.583	0.279	9.89E-133
Iglc3	1.79E-83	0.769403	0.828	0.694	5.57E-79
Cd24a	7.09E-105	0.74746	0.71	0.475	2.20E-100
Cd2	3.12E-118	0.734293	0.775	0.552	9.70E-114
Myadm	8.74E-143	0.719357	0.537	0.213	2.71E-138
Hspa8	5.42E-176	0.709709	0.935	0.822	1.68E-171
Arl5c	1.24E-72	0.706192	0.499	0.283	3.86E-68
Ucp2	1.13E-136	0.696751	0.804	0.575	3.51E-132
Igkc	3.64E-63	0.690625	0.953	0.961	1.13E-58
Ptma	4.46E-195	0.689157	0.961	0.905	1.39E-190
**CD21$^+$CD23$^+$**					
**genes**	**p value**	**Avg logFC**	**pct.1**	**pct.2**	**P value adjusted**
Fcer2a	4.35E-227	0.838628	0.653	0.253	1.35E-222
Ighd	1.45E-188	0.667697	0.827	0.619	4.50E-184
Neurl3	6.94E-130	0.600094	0.58	0.284	2.15E-125
Satb1	1.91E-145	0.571445	0.778	0.506	5.94E-141
Mef2c	6.60E-135	0.491009	0.934	0.842	2.05E-130
Fchsd2	2.67E-80	0.457044	0.706	0.545	8.30E-76
Gpr183	2.72E-52	0.43904	0.563	0.407	8.45E-48
Stap1	3.11E-68	0.428216	0.724	0.578	9.64E-64
Dmxl1	2.49E-54	0.417994	0.667	0.533	7.73E-50
Pgap1	1.52E-47	0.400051	0.535	0.393	4.73E-43
Lamb3	5.12E-44	0.384832	0.459	0.312	1.59E-39
Smad7	8.91E-45	0.378965	0.514	0.365	2.77E-40
Stat4	5.21E-40	0.357963	0.414	0.275	1.62E-35
Sell	5.37E-51	0.352693	0.617	0.485	1.67E-46
Zfp318	7.23E-32	0.301359	0.435	0.308	2.25E-27
Cr2	4.70E-30	0.2908	0.324	0.201	1.46E-25
H2-Ab1	1.04E-68	0.276937	0.972	0.876	3.23E-64

Vps37b	4.93E-19	0.271257	0.601	0.543	1.53E-14
Pxdc1	3.54E-42	0.271227	0.289	0.137	1.10E-37
H2-Aa	4.10E-55	0.269677	0.957	0.86	1.27E-50
**CD11b⁺**					
**genes**	**p value**	**Avg logFC**	**pct.1**	**pct.2**	**P value adjusted**
Ly6a	6.11E-166	1.063561	0.774	0.377	1.90E-161
Itgb1	5.09E-228	0.754993	0.621	0.142	1.58E-223
S100a4	5.35E-295	0.701682	0.393	0.031	1.66E-290
Odc1	1.05E-62	0.521991	0.626	0.321	3.25E-58
Traf1	1.06E-125	0.509379	0.563	0.172	3.28E-121
Tyrobp	6.82E-83	0.484692	0.509	0.185	2.12E-78
Zbtb20	3.47E-58	0.48393	0.632	0.329	1.08E-53
Ms4a6c	2.44E-138	0.475518	0.381	0.076	7.59E-134
Gimap4	4.05E-67	0.42572	0.746	0.416	1.26E-62
Rplp1	1.81E-57	0.424575	0.92	0.895	5.62E-53
Sp140	6.91E-61	0.409878	0.785	0.479	2.15E-56
Cybb	4.25E-62	0.402891	0.768	0.432	1.32E-57
Id2	1.14E-91	0.384671	0.387	0.11	3.53E-87
H2-Q7	9.53E-39	0.378988	0.752	0.497	2.96E-34
Cd9	9.98E-193	0.373671	0.442	0.073	3.10E-188
Ptpn18	1.45E-46	0.371551	0.799	0.641	4.50E-42
Fxyd5	9.39E-89	0.366216	0.67	0.286	2.92E-84
Cd44	1.71E-86	0.343201	0.553	0.204	5.30E-82
Ctla4	6.36E-134	0.336224	0.283	0.042	1.97E-129
Socs3	4.62E-59	0.334449	0.361	0.129	1.43E-54

Supplemental Table 3: Comparative analysis of B cell subsets ratio in the spleen from embryonic day 18 (E18) to adulthood (10 weeks old). Data were analyzed by 2-way ANOVA followed by Tukey's post-hoc test to correct for multiple comparisons.

	p value				
	**$IgM^{high}CD5^{+}$**	**$IgM^{high}CD5^{-}$**	**$CD21^{+}CD23^{+}$**	**$CD21^{-}CD23^{-}$**	**$CD21^{high}CD23^{-}$**
E18 vs. P1	0.1978	**0.0011**	0.9877	0.2007	n.a.
E18 vs. P3	**0.0120**	0.0702	**0.0452**	>0.9999	n.a.
E18 vs. P5	**<0.0001**	0.7884	**<0.0001**	**<0.0001**	0.6491
E18 vs. P7	**0.0167**	0.7222	**<0.0001**	**0.0101**	0.1352
E18 vs. P10	**0.0019**	0.9942	**0.0008**	**<0.0001**	**0.0355**
E18 vs. P14	**0.0003**	0.9965	**<0.0001**	**<0.0001**	0.0733
E18 vs. 3 weeks	>0.9999	0.0871	**0.0004**	**<0.0001**	**0.0049**
E18 vs. 5 weeks	>0.9999	0.9479	**<0.0001**	**<0.0001**	**0.0036**
E18 vs. 8 weeks	0.8241	>0.9999	**<0.0001**	**<0.0001**	**0.0035**
E18 vs. 10 weeks	0.4782	>0.9999	**<0.0001**	**<0.0001**	**0.0064**
P1 vs. P3	**0.0136**	0.9794	0.0690	0.4794	n.a.
P1 vs. P5	**0.0002**	**0.0032**	**<0.0001**	**<0.0001**	0.6491
P1 vs. P7	**0.0188**	**0.0068**	**<0.0001**	**0.0159**	0.1352
P1 vs. P10	**0.0023**	**0.0050**	**0.0010**	**<0.0001**	**0.0355**
P1 vs. P14	**0.0004**	**0.0013**	**<0.0001**	**0.0002**	0.0733
P1 vs. 3 weeks	**0.0300**	**0.0011**	**0.0005**	**0.0003**	**0.0049**
P1 vs. 5 weeks	**0.0003**	**0.0031**	**<0.0001**	**<0.0001**	**0.0036**
P1 vs. 8 weeks	0.2533	**0.0010**	**<0.0001**	**<0.0001**	**0.0035**
P1 vs. 10 weeks	0.2941	**0.0099**	**<0.0001**	**<0.0001**	**0.0064**
P3 vs. P5	**<0.0001**	0.0559	**0.0007**	**0.0014**	0.6491
P3 vs. P7	**0.0207**	0.1232	**<0.0001**	**0.0066**	0.1352
P3 vs. P10	**0.0046**	0.0924	**0.0008**	**0.0001**	**0.0355**
P3 vs. P14	0.8614	0.0860	**<0.0001**	**0.0005**	**0.0733**
P3 vs. 3 weeks	**0.0195**	**0.0353**	**0.0004**	**0.0003**	**0.0049**
P3 vs. 5 weeks	**0.0315**	0.0991	**<0.0001**	**<0.0001**	**0.0036**
P3 vs. 8 weeks	**0.0108**	0.0698	**<0.0001**	**<0.0001**	**0.0035**
P3 vs. 10 weeks	**0.0133**	0.0766	**<0.0001**	**<0.0001**	**0.0064**
P5 vs. P7	0.2758	0.2584	**0.0007**	0.0737	0.4820
P5 vs. P10	0.1105	0.1536	**0.0141**	**0.0381**	**0.0442**
P5 vs. P14	**<0.0001**	0.4148	**<0.0001**	0.8193	0.0922
P5 vs. 3 weeks	**<0.0001**	0.3698	**0.0016**	0.4448	**0.0040**
P5 vs. 5 weeks	**0.0002**	0.1093	**<0.0001**	**<0.0001**	**0.0037**
P5 vs. 8 weeks	**0.0001**	0.7957	**<0.0001**	**<0.0001**	**0.0035**
P5 vs. 10 weeks	**0.0002**	0.5694	**<0.0001**	**<0.0001**	**0.0065**
P7 vs. P10	**0.0467**	0.9129	0.4812	0.2930	0.1039
P7 vs. P14	**0.0253**	0.9841	**0.0019**	0.0992	0.1485
P7 vs. 3 weeks	**0.0193**	0.0813	**0.0058**	0.1356	**0.0064**
P7 vs. 5 weeks	**0.0203**	0.9905	**<0.0001**	**0.0287**	**0.0039**
P7 vs. 8 weeks	**0.0186**	0.7910	**<0.0001**	**0.0228**	**0.0036**
P7 vs. 10 weeks	**0.0189**	0.5104	**<0.0001**	**0.0179**	**0.0066**
P10 vs. P14	**0.0076**	>0.9999	**0.0409**	0.4011	0.9538
P10 vs. 3 weeks	**0.0027**	**0.0123**	**0.0052**	0.8328	**0.0340**
P10 vs. 5 weeks	**0.0031**	>0.9999	**<0.0001**	**<0.0001**	**0.0045**
P10 vs. 8 weeks	**0.0024**	0.9985	**<0.0001**	**<0.0001**	**0.0038**
P10 vs. 10 weeks	**0.0024**	0.7074	**<0.0001**	**<0.0001**	**0.0072**
P14 vs. 3 weeks	**0.0002**	0.0553	0.1046	0.9983	0.4474

P14 vs. 5 weeks	**0.0007**	>0.9999	**<0.0001**	**<0.0001**	**0.0046**
P14 vs. 8 weeks	**<0.0001**	0.9990	**<0.0001**	**<0.0001**	**0.0039**
P14 vs. 10 weeks	**0.0002**	0.9090	**<0.0001**	**<0.0001**	**0.0073**
3 weeks vs. 5 weeks	>0.9999	**0.0081**	**<0.0001**	**<0.0001**	**0.0060**
3 weeks vs. 8 weeks	0.3757	0.1255	**<0.0001**	**<0.0001**	**0.0044**
3 weeks vs. 10 weeks	0.0849	**0.0457**	**<0.0001**	**<0.0001**	**0.0084**
5 weeks vs. 8 weeks	0.1140	0.9768	**0.0170**	**0.0004**	0.0829
5 weeks vs. 10 weeks	**0.0023**	0.5268	**<0.0001**	**<0.0001**	0.3702
8 weeks vs. 10 weeks	0.9641	>0.9999	**0.0456**	**0.0007**	0.9797

*n.a. – not applicable: Mean Difference = 0.

Supplemental Table 4: Comparative analysis of B cell subsets ratio in the heart. blood. spleen. lung. and liver from P1 to 5 weeks of age. Data were analyzed by 2-way ANOVA followed by Tukey's post-hoc test to correct for multiple comparisons.

	***p* value**				
**$CD5^{+}IgM^{high}$**	**P1**	**P10**	**P14**	**P21**	**5 weeks**
Heart vs. Blood	0.9952	0.8659	0.1571	0.5161	0.8766
Heart vs. Lung	0.3451	**<0.0001**	**<0.0001**	>0.9999	0.1441
Heart vs. Liver	0.9414	**<0.0001**	**<0.0001**	0.9938	0.9996
Heart vs. Spleen	0.9913	0.9997	**0.0005**	0.741	0.1741
Blood vs. Lung	0.581	**<0.0001**	**<0.0001**	0.5319	**0.0123**
Blood vs. Liver	0.7815	**<0.0001**	0.1657	0.7588	0.8041
Blood vs. Spleen	>0.9999	0.768	0.4492	0.0576	**0.0161**
Lung vs. Liver	0.0744	**<0.0001**	**<0.0001**	0.9915	0.2792
Lung vs. Spleen	0.623	**<0.0001**	**<0.0001**	0.8117	>0.9999
Liver vs. Spleen	0.7446	**<0.0001**	0.9722	0.4815	0.3228
**$CD5^{-}IgM^{high}$**	**P1**	**P10**	**P14**	**P21**	**5 weeks**
Heart vs. Blood	**0.0310**	>0.9999	0.9998	0.2612	0.9991
Heart vs. Lung	0.4652	0.9082	**0.0243**	0.9889	**<0.0001**
Heart vs. Liver	0.3838	**0.0028**	**0.0030**	0.3645	0.1479
Heart vs. Spleen	**<0.0001**	0.5473	0.9769	0.3228	**0.0074**
Blood vs. Lung	0.6900	0.9480	**0.0244**	0.1297	**<0.0001**
Blood vs. Liver	**<0.0001**	**0.0018**	**0.0105**	0.9981	0.0867
Blood vs. Spleen	**0.0139**	0.6328	0.9501	**0.0022**	**0.0160**
Lung vs. Liver	**0.0073**	**0.0001**	**<0.0001**	0.1872	**<0.0001**
Lung vs. Spleen	**0.0002**	0.9643	0.1082	0.6842	0.4597
Liver vs. Spleen	**<0.0001**	**<0.0001**	**0.0004**	**0.0029**	**<0.0001**
**$CD21^{+}CD23^{+}$**	**P1**	**P10**	**P14**	**P21**	**5 weeks**
Heart vs. Blood	0.9995	0.1196	0.6867	**0.0001**	0.7120
Heart vs. Lung	0.9336	0.4350	0.6495	0.7056	0.0913
Heart vs. Liver	0.9978	**<0.0001**	**0.0001**	0.1036	**0.0383**
Heart vs. Spleen	0.9778	0.6524	0.9991	**0.0121**	0.1285
Blood vs. Lung	0.8545	0.9536	>0.9999	**0.0158**	**0.0023**
Blood vs. Liver	>0.9999	**0.0429**	**0.0265**	0.1514	0.4464
Blood vs. Spleen	0.9321	0.8295	0.8199	**<0.0001**	**0.0038**
Lung vs. Liver	0.8050	**0.0053**	**0.0148**	0.8257	**<0.0001**
Lung vs. Spleen	0.9996	0.9970	0.7964	**0.0003**	0.9999
Liver vs. Spleen	0.8984	**0.0017**	**0.0003**	**<0.0001**	**<0.0001**
**$CD21^{-}CD23^{-}$**	**P1**	**P10**	**P14**	**P21**	**5 weeks**
Heart vs. Blood	**<0.0001**	0.6507	0.9999	**<0.0001**	0.9699
Heart vs. Lung	**<0.0001**	**0.0036**	**0.0069**	0.9988	0.7935
Heart vs. Liver	0.0943	**<0.0001**	**<0.0001**	**<0.0001**	0.7570
Heart vs. Spleen	**<0.0001**	0.1109	**0.0013**	0.9994	0.9646
Blood vs. Lung	**<0.0001**	**<0.0001**	**0.0080**	**<0.0001**	0.9877
Blood vs. Liver	**<0.0001**	**<0.0001**	**<0.0001**	0.1720	0.3888
Blood vs. Spleen	**<0.0001**	0.8140	**0.0048**	**0.0001**	0.6987
Lung vs. Liver	**<0.0001**	**<0.0001**	**<0.0001**	**<0.0001**	0.1706
Lung vs. Spleen	0.7596	**<0.0001**	**<0.0001**	0.9889	0.3894
Liver vs. Spleen	**<0.0001**	**<0.0001**	**<0.0001**	**<0.0001**	0.9793

Supplemental Table 5: List of genes differentially expressed in the heart. blood. liver and lung associated B cells (FDR<0.05). Genes were filtered by Seruat as logFC<-0.25 and logFC>0.25.

Tissue	Gene	P value	Avg logFC	pct.1	pct.2	FDR
**Heart**	Tsc22d3	1.80E-39	0.51096	0.917	0.887	5.60E-35
**Heart**	H3f3b	2.32E-32	0.279229	0.965	0.962	7.22E-28
**Heart**	Ltb	1.03E-31	-0.4771	0.553	0.758	3.21E-27
**Heart**	Rsrp1	1.85E-28	0.265148	0.95	0.92	5.73E-24
**Heart**	Sertad1	1.34E-27	0.37262	0.761	0.61	4.16E-23
**Heart**	Ppp1r15a	2.84E-21	0.309911	0.723	0.552	8.83E-17
**Heart**	Hspa5	1.22E-19	0.260398	0.907	0.853	3.79E-15
**Heart**	Fam107b	1.64E-19	0.278719	0.893	0.89	5.09E-15
**Heart**	Hvcn1	9.35E-19	0.383688	0.738	0.623	2.90E-14
**Heart**	Ccr7	9.85E-17	-0.29137	0.84	0.922	3.06E-12
**Heart**	Dusp5	8.50E-12	0.388447	0.444	0.316	2.64E-07
**Heart**	Slfn2	2.45E-11	-0.26319	0.467	0.615	7.62E-07
**Heart**	Txnip	3.84E-11	0.267175	0.82	0.756	1.19E-06
**Heart**	Plk2	4.57E-09	0.296815	0.285	0.181	1.42E-04
**Heart**	Zfp36l2	5.35E-08	0.277817	0.756	0.718	0.00166
**Blood**	H3f3b	8.86E-96	-0.4975	0.96	0.963	2.75E-91
**Blood**	Slfn2	8.75E-77	0.614287	0.808	0.499	2.72E-72
**Blood**	Nr4a1	1.07E-75	-0.84732	0.667	0.852	3.31E-71
**Blood**	Junb	1.12E-75	-0.57947	0.952	0.967	3.47E-71
**Blood**	Hspa8	5.92E-68	-0.46816	0.942	0.952	1.84E-63
**Blood**	Eif1	4.55E-62	-0.31926	0.959	0.96	1.41E-57
**Blood**	Ltb	2.22E-61	0.559167	0.865	0.658	6.91E-57
**Blood**	Cd79b	2.51E-58	0.349156	0.969	0.922	7.79E-54
**Blood**	Hspa5	1.17E-55	-0.47158	0.781	0.896	3.63E-51
**Blood**	Vps37b	7.50E-55	-0.66371	0.608	0.804	2.33E-50
**Blood**	Rsrp1	1.16E-52	-0.40427	0.888	0.94	3.62E-48
**Blood**	Fos	1.73E-50	-0.75049	0.095	0.377	5.39E-46
**Blood**	Ftl1	9.72E-50	-0.38472	0.943	0.944	3.02E-45
**Blood**	Crem	1.28E-48	-0.55081	0.259	0.544	3.97E-44
**Blood**	Ubc	3.78E-48	-0.37127	0.926	0.936	1.17E-43
**Blood**	Ppp1r15a	4.48E-47	-0.52638	0.399	0.66	1.39E-42
**Blood**	Tob2	6.16E-47	-0.5052	0.471	0.709	1.91E-42
**Blood**	Sertad1	1.29E-44	-0.49682	0.481	0.702	3.99E-40
**Blood**	Dusp1	7.40E-44	-0.64697	0.253	0.518	2.30E-39
**Blood**	Actg1	1.16E-36	-0.38401	0.907	0.939	3.61E-32
**Blood**	Srgn	1.35E-35	-0.3406	0.932	0.939	4.19E-31
**Blood**	Pim1	5.44E-34	-0.45582	0.557	0.73	1.69E-29
**Blood**	Rel	5.07E-32	-0.43411	0.631	0.787	1.58E-27
**Blood**	Hsp90ab1	1.73E-31	-0.32178	0.933	0.947	5.36E-27
**Blood**	Kdm6b	2.37E-30	-0.3896	0.298	0.528	7.37E-26
**Blood**	Dnaja1	3.54E-30	-0.38298	0.578	0.745	1.10E-25
**Blood**	Il16	3.93E-30	0.334229	0.523	0.313	1.22E-25

**Blood**	Cebpb	4.77E-30	-0.42578	0.197	0.419	1.48E-25
**Blood**	Myc	9.11E-30	-0.52867	0.052	0.237	2.83E-25
**Blood**	Klf4	9.93E-30	-0.3727	0.081	0.276	3.09E-25
**Blood**	Tsc22d3	1.01E-29	-0.46513	0.875	0.9	3.14E-25
**Blood**	Hsp90aa1	1.80E-29	-0.39176	0.586	0.743	5.59E-25
**Blood**	Cytip	7.60E-29	-0.31311	0.892	0.928	2.36E-24
**Blood**	Arhgap45	9.25E-29	0.263477	0.939	0.853	2.87E-24
**Blood**	Litaf	2.07E-28	-0.43192	0.392	0.588	6.42E-24
**Blood**	Fosb	2.26E-27	-0.32846	0.054	0.228	7.02E-23
**Blood**	Nfkbid	3.24E-27	-0.39184	0.364	0.571	1.01E-22
**Blood**	Dusp5	1.85E-26	-0.45738	0.199	0.398	5.76E-22
**Blood**	Dynll1	4.45E-26	-0.33004	0.735	0.837	1.38E-21
**Blood**	Fam107b	5.85E-25	-0.31526	0.855	0.904	1.82E-20
**Blood**	Tnfrsf13c	3.20E-24	0.285198	0.621	0.43	9.94E-20
**Blood**	Gem	5.71E-24	-0.38636	0.223	0.416	1.77E-19
**Blood**	Iglc3	7.73E-24	0.26969	0.828	0.685	2.40E-19
**Blood**	Ifngr2	2.71E-23	0.284059	0.375	0.202	8.42E-19
**Blood**	Ifrd1	3.77E-23	-0.30587	0.27	0.465	1.17E-18
**Blood**	Plaur	3.97E-23	-0.41226	0.676	0.76	1.23E-18
**Blood**	Plk2	4.28E-23	-0.46589	0.084	0.248	1.33E-18
**Blood**	Ier2	1.92E-20	-0.27193	0.865	0.898	5.97E-16
**Blood**	Per1	1.48E-19	-0.27087	0.243	0.422	4.58E-15
**Blood**	Tob1	1.17E-18	-0.30751	0.292	0.464	3.63E-14
**Blood**	Clk1	1.48E-18	-0.25864	0.56	0.717	4.58E-14
**Blood**	Got1	1.51E-18	-0.33578	0.275	0.439	4.69E-14
**Blood**	Ppp1r18	1.90E-18	0.26068	0.714	0.588	5.91E-14
**Blood**	Wsb1	3.26E-18	-0.27466	0.452	0.614	1.01E-13
**Blood**	Cd86	2.45E-17	-0.28552	0.175	0.332	7.61E-13
**Blood**	Tra2a	3.70E-17	-0.29084	0.335	0.497	1.15E-12
**Blood**	Sik1	1.95E-16	-0.27574	0.237	0.39	6.05E-12
**Blood**	Gimap1	1.97E-16	0.252142	0.652	0.515	6.12E-12
**Blood**	Rilpl2	3.71E-16	-0.41793	0.359	0.491	1.15E-11
**Blood**	Cd24a	5.89E-16	-0.26179	0.612	0.73	1.83E-11
**Blood**	Brd2	1.53E-15	-0.25055	0.69	0.783	4.76E-11
**Blood**	Tuba1c	1.77E-15	-0.27681	0.476	0.598	5.48E-11
**Blood**	Dnajb9	1.90E-15	-0.25202	0.209	0.358	5.89E-11
**Blood**	Hvcn1	3.13E-15	-0.3098	0.552	0.683	9.72E-11
**Blood**	Cyth1	3.49E-15	-0.27233	0.543	0.669	1.09E-10
**Blood**	Klf6	5.47E-15	-0.3303	0.299	0.434	1.70E-10
**Blood**	Satb1	8.06E-14	-0.25392	0.369	0.511	2.50E-09
**Blood**	Trim25	1.67E-12	-0.2787	0.462	0.575	5.19E-08
**Blood**	Jun	2.70E-12	-0.31286	0.147	0.267	8.40E-08
**Blood**	Gm26532	3.39E-12	-0.27249	0.217	0.342	1.05E-07
**Blood**	Elmsan1	5.62E-12	-0.25504	0.31	0.431	1.75E-07
**Blood**	Bambi	8.07E-11	-0.25115	0.093	0.194	2.51E-06
**Blood**	Cd83	2.72E-08	-0.25628	0.667	0.704	8.44E-04

**Blood**	Rrad	7.50E-08	-0.26229	0.33	0.423	0.002329
**Blood**	Gm26917	1.09E-06	-0.25718	0.452	0.54	0.0338
**Liver**	Rps24	3.36E-59	0.250408	0.95	0.952	1.04E-54
**Liver**	Uba52	2.70E-57	0.259568	0.95	0.956	8.40E-53
**Liver**	Ccr7	7.59E-45	0.50543	0.931	0.897	2.36E-40
**Liver**	Cd83	9.13E-45	0.548889	0.808	0.66	2.84E-40
**Liver**	Srgn	3.34E-36	0.317417	0.936	0.937	1.04E-31
**Liver**	mt-Atp8	6.27E-34	0.289551	1	1	1.95E-29
**Liver**	mt-Nd4l	1.67E-30	0.310218	1	0.999	5.17E-26
**Liver**	Hspa8	1.98E-28	0.264203	0.952	0.948	6.15E-24
**Liver**	Stk17b	5.62E-28	0.300251	0.948	0.942	1.74E-23
**Liver**	Plaur	3.21E-27	0.415535	0.809	0.716	9.97E-23
**Liver**	Cxcr5	2.69E-26	0.409898	0.606	0.469	8.35E-22
**Liver**	Junb	1.16E-24	0.280433	0.973	0.96	3.61E-20
**Liver**	Nr4a1	3.92E-24	0.378965	0.891	0.775	1.22E-19
**Liver**	mt-Co2	1.49E-23	0.256408	1	1	4.62E-19
**Liver**	Nfkbid	1.11E-20	0.368216	0.62	0.483	3.46E-16
**Liver**	Rel	2.40E-20	0.379812	0.792	0.73	7.45E-16
**Liver**	Jund	5.14E-20	0.296905	0.867	0.843	1.60E-15
**Liver**	mt-Nd2	1.36E-19	0.269796	0.98	0.985	4.22E-15
**Liver**	Fosb	7.16E-19	0.308426	0.289	0.148	2.22E-14
**Liver**	Gimap6	9.22E-19	0.293027	0.856	0.83	2.86E-14
**Liver**	Bcl2a1b	9.01E-17	0.390127	0.339	0.21	2.80E-12
**Liver**	Gpr183	1.15E-16	0.380672	0.431	0.301	3.57E-12
**Liver**	Txnip	2.61E-16	0.256408	0.816	0.756	8.10E-12
**Liver**	Smim14	3.22E-14	0.259431	0.747	0.712	9.99E-10
**Liver**	Btg1	3.25E-14	0.263282	0.933	0.926	1.01E-09
**Liver**	Dennd4a	5.21E-14	0.320645	0.681	0.621	1.62E-09
**Liver**	Fos	2.68E-13	0.383479	0.4	0.27	8.31E-09
**Liver**	Lbh	1.41E-12	0.276509	0.475	0.372	4.37E-08
**Liver**	Sell	2.87E-12	0.266596	0.7	0.639	8.91E-08
**Liver**	Gimap3	3.92E-12	0.264901	0.602	0.517	1.22E-07
**Liver**	Adgre5	7.04E-12	0.286508	0.667	0.598	2.19E-07
**Liver**	Myc	1.70E-10	0.547639	0.262	0.164	5.27E-06
**Liver**	4930523C07Rik	2.77E-10	0.253586	0.641	0.598	8.60E-06
**Liver**	Rilpl2	8.31E-10	0.326314	0.517	0.437	2.58E-05
**Liver**	Irf4	1.20E-09	0.272905	0.308	0.215	3.72E-05
**Liver**	Vps37b	3.47E-09	0.251064	0.784	0.74	1.08E-04
**Liver**	Rasgef1b	1.28E-08	0.270129	0.353	0.272	3.98E-04
**Liver**	Klf6	4.39E-07	0.286535	0.445	0.383	0.013632
**Lung**	Txnip	1.19E-50	-0.52631	0.67	0.809	3.69E-46
**Lung**	Arhgdib	7.70E-32	-0.26024	0.931	0.928	2.39E-27
**Lung**	Shisa5	5.96E-31	-0.31016	0.883	0.909	1.85E-26
**Lung**	mt-Nd4l	5.54E-30	-0.2663	0.998	1	1.72E-25
**Lung**	Ypel3	8.39E-25	-0.3092	0.732	0.778	2.61E-20
**Lung**	mt-Nd2	2.99E-22	-0.25025	0.984	0.984	9.29E-18

**Lung**	Slfn2	4.72E-22	-0.42303	0.496	0.619	1.47E-17
**Lung**	Gimap6	6.86E-22	-0.29175	0.79	0.854	2.13E-17
**Lung**	Gimap1	1.37E-18	-0.30793	0.483	0.58	4.27E-14
**Lung**	Ltb	1.96E-17	-0.34838	0.666	0.734	6.07E-13
**Lung**	Gimap3	9.19E-17	-0.31288	0.47	0.563	2.85E-12
**Lung**	Iglc3	1.52E-14	-0.28554	0.705	0.732	4.71E-10
**Lung**	Ucp2	1.07E-13	-0.25267	0.782	0.799	3.31E-09
**Lung**	Fcer2a	2.62E-12	-0.29271	0.666	0.725	8.13E-08

Supplemental Table 6: KEGG pathway analysis of genes differentially expressed between blood and organ-associated B cells. Genes were analyzed for Immune System and Signal Transduction Pathways.

Gene Set Name	# Genes in Gene Set (K)	# Genes in Overlap (k)	k/K	p-value
KEGG_MAPK_SIGNALING_PATHWAY	267	6	0.0225	1.1E-5
KEGG_B_CELL_RECEPTOR_SIGNALING_PATHWAY	75	3	0.0400	3.82E-4
KEGG_ANTIGEN_PROCESSING_AND_PRESENTATION	88	3	0.0341	6.1E-4
KEGG_TOLL_LIKE_RECEPTOR_SIGNALING_PATHWAY	102	3	0.0294	9.36E-4

Supplemental Table 7: Gene list of cluster 0 markers.

Gene name	P value	avg_logFC	pct.1	pct.2	Adj. p value
Nr4a1	8.62E-96	0.610281	0.963	0.761	2.68E-91
Junb	3.4E-103	0.492133	0.994	0.955	1.06E-98
Fos	8.52E-28	0.468779	0.449	0.286	2.65E-23
Dusp1	2.36E-39	0.438132	0.618	0.413	7.33E-35
Ppp1r15a	2.04E-51	0.438004	0.763	0.561	6.34E-47
Sertad1	7.26E-57	0.437974	0.826	0.613	2.25E-52
Plaur	6.32E-48	0.386336	0.899	0.703	1.96E-43
Vps37b	4.07E-43	0.383584	0.871	0.736	1.26E-38
Ier2	5.31E-47	0.336228	0.964	0.87	1.65E-42
H3f3b	2.66E-69	0.32224	0.999	0.951	8.25E-65
Plk2	8.86E-12	0.303037	0.294	0.201	2.75E-07
Cebpb	9.58E-24	0.298806	0.516	0.36	2.98E-19
Ubc	1.8E-47	0.291393	0.986	0.917	5.59E-43
Hspa5	1.13E-39	0.289261	0.952	0.844	3.51E-35
Pnrc1	1.9E-40	0.283858	0.98	0.903	5.9E-36
Eif1	4.22E-64	0.282373	0.999	0.946	1.31E-59
Litaf	1.74E-16	0.270325	0.636	0.542	5.41E-12
Hspa8	3.67E-30	0.263399	0.997	0.935	1.14E-25
Tuba1c	3.94E-19	0.263097	0.659	0.572	1.22E-14
Rrad	1.21E-10	0.261136	0.491	0.409	3.77E-06
Hs3st1	7.44E-14	0.251497	0.412	0.313	2.31E-09
Blk	1.02E-18	-0.25126	0.623	0.74	3.16E-14
Samhd1	4.58E-19	-0.25499	0.716	0.803	1.42E-14
mt-Nd5	8.68E-20	-0.25617	0.928	0.961	2.69E-15
Foxp1	2.42E-19	-0.25699	0.923	0.926	7.51E-15
Arpc5l	1.39E-22	-0.25938	0.384	0.564	4.32E-18
Tcf3	1.52E-19	-0.2602	0.509	0.671	4.73E-15
Ikzf3	7.27E-19	-0.26023	0.453	0.618	2.26E-14
Gm30211	1.07E-08	-0.26027	0.397	0.492	0.000332
Ifi27l2a	2.94E-08	-0.26094	0.564	0.641	0.000913
Arhgap45	2.33E-28	-0.26776	0.839	0.913	7.25E-24
Pld4	1.77E-17	-0.27788	0.282	0.438	5.5E-13
Hck	2.19E-22	-0.27822	0.128	0.282	6.79E-18
mt-Atp8	3.3E-28	-0.27952	1	1	1.03E-23
Pou2f2	8.74E-23	-0.28255	0.502	0.651	2.72E-18
mt-Co1	2.24E-42	-0.28654	1	1	6.95E-38
mt-Co2	1.32E-46	-0.29202	1	1	4.11E-42
mt-Co3	3.14E-41	-0.29514	1	1	9.76E-37
Iglc3	1.33E-24	-0.30279	0.687	0.777	4.14E-20
Siglecg	1.5E-24	-0.30608	0.54	0.71	4.65E-20
Ahnak	6.5E-16	-0.30851	0.186	0.317	2.02E-11
Ucp2	5.31E-25	-0.31292	0.782	0.829	1.65E-20

mt-Nd4l	3.58E-45	-0.31824	0.997	1	1.11E-40
Ptprcap	4.24E-35	-0.31833	0.683	0.808	1.32E-30
mt-Cytb	4.79E-47	-0.32138	1	1	1.49E-42
Tagln2	2.53E-13	-0.32167	0.553	0.664	7.85E-09
Cnp	5.96E-29	-0.32296	0.552	0.724	1.85E-24
Ptpn6	4.49E-24	-0.3329	0.525	0.694	1.39E-19
Fam129c	4.46E-36	-0.33974	0.07	0.258	1.38E-31
Vim	6.69E-15	-0.34997	0.638	0.728	2.08E-10
Dnajc7	1.08E-21	-0.35351	0.398	0.572	3.37E-17
Spib	3.99E-23	-0.37505	0.353	0.536	1.24E-18
Vpreb3	2.41E-12	-0.38554	0.434	0.546	7.5E-08
Iglc1	2.04E-40	-0.39631	0.115	0.332	6.34E-36
S100a6	3.72E-10	-0.40707	0.046	0.114	1.15E-05
Iglc2	1.85E-27	-0.41949	0.681	0.777	5.73E-23
Crip1	3.84E-16	-0.42509	0.732	0.792	1.19E-11
Tnfrsf13c	1.61E-34	-0.42599	0.363	0.58	4.99E-30
Ltb	1.74E-42	-0.45077	0.635	0.791	5.4E-38
Ighm	1.99E-62	-0.46319	0.851	0.907	6.17E-58
Ms4a1	1.53E-34	-0.4731	0.854	0.89	4.74E-30
Ly6d	2E-33	-0.52303	0.91	0.892	6.22E-29
Slfn2	5.62E-63	-0.56886	0.431	0.696	1.75E-58
Cd79b	4.1E-114	-0.66661	0.943	0.942	1.3E-109

Supplemental Table 8: KEGG pathway analysis of genes differentially expressed in cluster 0. Genes were analyzed for Immune System and Energy Metabolism pathways.

Gene Set Name	# Genes in Gene Set (K)	# Genes in Overlap (k)	k/K	p-value
KEGG_OXIDATIVE_PHOSPHORYLATION	131	7	0.0534	1.27E-9
KEGG_B_CELL_RECEPTOR_SIGNALING_PATHWAY	75	3	0.0400	2.1E-4

Supplemental Table 9: Antibodies used in flow cytometry experiments.

Specificity	Brand	Clone	Fluorophore	Cat #
CD45	BioLegend	30-F11	PerCP-Cy5	103132
CD19	BioLegend	1D3/CD19	APC	152410
IgD	BioLegend	11-16c.2a	BV605	405727
CD11b	BioLegend	M1/70	PE	101208
IgM	BioLegend	RMM-1	Alexa 488	406522
CD5	BioLegend	53-7.3	PE-Cy7	100622
CD21/CD35	BioLegend	7E9	BV421	123422
CD23	BioLegend	B3B4	APC-Cy7	101630

Supplemental Table 10: Oligo-tagged antibodies used for scRNA seq analysis.

Specificity	Brand	Clone	Catalog#
TotalSeqTM-A0014 anti-mouse/human CD11b	BioLegend	M1/70	101265
TotalSeqTM-A0108 anti-mouse CD23	BioLegend	B3B4	101635
TotalSeqTM-A0107 anti-mouse CD21/CD35 (CR2/CR1)	BioLegend	7E9	123427
TotalSeqTM-C0301 anti-mouse Hashtag 1	BioLegend	M1/42	155861
TotalSeqTM-C0302 anti-mouse Hashtag 2	BioLegend	M1/42	155863
TotalSeqTM-C0303 anti-mouse Hashtag 3	BioLegend	M1/42	155865
TotalSeqTM-C0304 anti-mouse Hashtag 4	BioLegend	M1/42	155867

Supplemental Movies Legends

Supplemental Video 1: Z-stack of a myocardial section of CD19-tdTomato mouse heart at embryonic day 18 (E18). A B cell is visible in the intravascular space. Red: B cell, Green: CD31, Blue: DAPI

Supplemental Video 2: Z-stack of a myocardial section of CD19-tdTomato mouse heart at post-neonatal day 7 (P7). B cells are visible in the intravascular space. Red: B cell, Green: CD31, Blue: DAPI.

Supplemental Video 3: Z-stack of a myocardial section of CD19-tdTomato mouse heart at post-neonatal day 14 (P14). B cells are visible in the intravascular space. Red: B cell, Green: CD31, Blue: DAPI.

Supplemental Video 4: Z-stack of a myocardial section of CD19-tdTomato mouse heart at 5 weeks of age. B cells are visible in the intravascular space. Red: B cell, Green: CD31, Blue: DAPI.

Supplemental Video 5: Z-stack of a section of CD19-tdTomato mouse liver at post-neonatal day 7 (P7). B cells are visible in the intravascular space. Red: B cell, Green: CD31, Blue: DAPI.

Supplemental Video 6: Z-stack of a section of CD19-tdTomato mouse liver at post-neonatal day 14 (P14). B cells are visible in the intravascular space. Red: B cell, Green: CD31, Blue: DAPI.

Supplemental Video 7: Z-stack of a section of CD19-tdTomato mouse liver at 5 weeks of age. B cells are visible in the intravascular space. Red: B cell, Green: CD31, Blue: DAPI.

Supplemental Video 8: Z-stack of a section of CD19-tdTomato mouse lung at post-neonatal day 7 (P7). B cells are visible in the intravascular space. Red: B cell, Green: CD31, Blue: DAPI.

Supplemental Video 9: Z-stack of a section of CD19-tdTomato mouse lung at post-neonatal day 14 (P14). B cells are visible in the intravascular space. Red: B cell, Green: CD31, Blue: DAPI.

Supplemental Video 9: Z-stack of a section of CD19-tdTomato mouse lung at 5 weeks of age. B cells are visible in the intravascular space. Red: B cell, Green: CD31, Blue: DAPI.

Supplemental Video 11: Intravital microscopy of B cells in the lung of adult CD19-tdTomato mice. Lung B cells are mostly intravascular. Some B cells are seen rapidly flowing through blood vessels, while some other B cells move slowly or even paused in the lung endotelial. Blood vessels were marked with intravenous injection of high molecular weight fluorescent dextran. B cells are shown in green; Blood vessels in red; DAPI: blue.

Supplemental Video 12: Intravital microscopy of B cells in the lung of adult CD19-tdTomato mice. Blood vessels were marked with intravenous injection of high molecular weight fluorescent dextran. B cells are shown in green; Blood vessels in red; DAPI: blue.

Supplemental Video 13: Intravital microscopy of B cells in the lung of adult CD19-tdTomato mice. In some regions of the lung, B cells were found stopped or moving slowly throught the endotelium. Blood vessels were marked with intravenous injection of high molecular weight fluorescent dextran. B cells are shown in green; Blood vessels in red; DAPI: blue.

Reference

1. Adamo L. Rocha-Resende C. Lin CY. Evans S. Williams J. Dun H. et al. Myocardial B cells are a subset of circulating lymphocytes with delayed transit through the heart. *JCI Insight.* 2020;5(3).
